# Microclimate effects and outdoor thermal comfort of green roof types in hot and dry climates: Modelling in the historic city of Yazd, Iran

**DOI:** 10.1371/journal.pone.0325494

**Published:** 2025-06-10

**Authors:** Zahra Karimian, Sara Mahdizadeh

**Affiliations:** 1 Department of Horticultural Science and Landscape Architecture, Faculty of Agriculture, Ferdowsi University of Mashhad, Mashhad, Iran; 2 School of Civil Engineering, University of Leeds, Leeds, United Kingdom; Changan University: Chang'an University, CHINA

## Abstract

In hot and arid climates, developing green roofs to improve the microclimate and thermal comfort faces challenges due to water scarcity and harsh climate conditions. To evaluate the effect of green roof types on microclimate parameters and thermal comfort, a simulation was conducted in Yazd, Iran, using the ENVI-met model. Three scenarios—intensive green roofs, extensive green roofs, and roofs without vegetation—were simulated using meteorological data from 7:00 am to 6:00 PM during the hottest period of the year. Desert-adapted plant species were included in two green roof types. The model outputs indicated that, compared to extensive green roofs and roofs without vegetation, intensive green roofs resulted in lower air temperature, mean radiant temperature, and longwave radiation. They also led to higher wind speed and relative humidity, contributing to more desirable thermal comfort. Extensive green roofs and roofs without vegetation generally showed no significant differences in the measured microclimatic parameters or thermal comfort index. As suggested by the findings of this study, intensive green roofs demonstrated superior performance in enhancing thermal comfort compared to extensive green roofs. However, during the hottest period of the year and within the measured hours, all three scenarios were classified as ‘very hot’ (PMV = 5.03) and ‘hot’ (PMV = 3.2), experiencing strong to extreme heat stress, respectively. The measured hours and distance from the roofs affected the microclimatic parameters and thermal comfort, with the intensive green roof showing the most favorable thermal comfort condition (PMV = 0.18) during 7:00–9:00 am, perceived as comfortable with no thermal stress. However, the microclimatic improvements and thermal comfort enhancements resulting from the simulated green roofs in the surrounding environment) were not significant. Considering the outcomes alongside the severe climatic conditions prevalent in the city of Yazd, characterized by high temperatures, intense radiation during the summer, and extreme water scarcity, the proposition for the construction and development of green roofs in this region is not advisable. Although green roofs aim to ameliorate the microclimate and improve thermal comfort during hot periods, their effectiveness under such harsh conditions remains limited.

## 1. Introduction

Climate change, often associated with extreme compound events such as droughts and heatwaves, is particularly evident in arid and semi-arid regions [1]. These regions cover approximately 41% of the Earth’s land surface and are home to around 2.5 billion people [2]. Their extent is expected to increase as a result of climate change. In addition to natural climate variability, urban environments are reported to be 3.5°C–4.5°C warmer than surrounding areas, with projections indicating an average temperature increase of approximately 1°C per decade [3]. As a result, urban landscape planning and management, particularly in arid and semi-arid regions, face significant challenges due to high summer temperatures, intense sunlight, drought, and water scarcity [4].

Improving the availability of adequate green space per capita, given its numerous health and environmental benefits, is considered a key objective in urban landscape planning [5]. Plants, as essential living components of the urban landscape, are profoundly influenced by climatic conditions through their interactions with the environment. Factors such as water scarcity, drought, high temperatures, and radiation present significant challenges to the growth, establishment, and survival of plants, particularly those not adapted to such conditions [6]. Consequently, providing sufficient green space per capita in arid urban landscapes, especially in densely populated cities, remains a considerable challenge. This issue is further compounded by limited land availability and the presence of historical and cultural heritage sites, which restrict the horizontal expansion of vegetation cover.

The implementation of vertical infrastructures, such as green roofs and walls, is widely recognized as an effective strategy to increase vegetative coverage and green space per capita in densely populated cities worldwide [7]. Among these, green roofs are gaining prominence due to their numerous environmental benefits, including mitigating urban heat island effects [8], providing suitable habitats for biodiversity [9], managing urban runoff [10], and improving air and water quality [11,12].

A key function of green roofs is their ability to improve microclimates and enhance thermal comfort, particularly during warm seasons [13–17]. Numerous studies have highlighted that vegetative coverage, through mechanisms such as shading, increased surface albedo, evapotranspiration, and the modification of wind patterns, plays a significant role in microclimate improvement [18–22]. Green roofs are categorized into two main types: extensive and intensive [23]. Extensive green roofs, characterized by shallow soil depth and low-growing vegetation, require minimal maintenance and can often function without a permanent irrigation system. In contrast, intensive green roofs, which have a deeper substrate capable of supporting larger plants, require systematic maintenance and irrigation [23,24]. Semi-intensive green roofs represent another category, exhibiting characteristics that are intermediate between the extensive and intensive classifications [24].

An important aspect of investigating and evaluating the impact of vegetation on the quantity and quality of human life in various environments is human thermal comfort. Thermal comfort refers to a psychological state of satisfaction with the thermal environment, determined through subjective assessment [25]. The primary microclimatic factors essential for assessing thermal comfort include air temperature, relative humidity, air movement, and radiation [26–28]. Due to the presence of vegetation and the materials used in their construction, green roofs influence these microclimatic factors and enhance roof albedo, resulting in reduced temperatures and improved thermal comfort within buildings and their surrounding environments [23,29–32].

Most existing research focuses on the impact of green roofs on microclimates and thermal comfort in temperate regions, primarily in Europe and North America. These studies indicate that implementing green roofs in arid and semi-arid regions presents significant challenges [33,34]. Reports from hot and arid regions suggest that adequate irrigation of vegetation is crucial for improving thermal comfort [35,36]. In such climates, green roofs with more compact and deeper systems tend to perform better due to their enhanced water retention capacity and improved soil moisture following rainfall events [37]. Field data analysis and modeling have demonstrated the positive effects of green roofs in reducing air temperature, lowering energy consumption, and improving thermal comfort in several hot and dry cities, including Cairo, Egypt [38], Amman, Jordan [39], and Mashhad, Iran [40].

The harsh climatic conditions prevalent in arid and semi-arid regions, especially in densely populated cities include low precipitation, high temperatures, intense solar radiation, persistent winds, significant temperature fluctuations between day and night, and low humidity. These factors present substantial challenges to achieving human thermal comfort [1,4,33,34].

The primary aim of this research is to conduct simulations and comparative analyses of microclimatic parameters and thermal comfort using two distinct types of green roofs, extensive and intensive, implemented in Yazd, a desert city in Iran. The study addresses the following specific objectives: 1. To evaluate the impact of green roofs on key microclimatic variables, namely temperature, relative humidity, radiation, and wind speed, and their influence on human thermal comfort during the warm seasons in a desert environment. 2. To identify the most effective type of green roof (extensive or intensive) in modifying microclimatic conditions and enhancing human thermal comfort in Yazd, a representative desert city. 3. To assess the feasibility and potential benefits of incorporating green roofs into urban landscape planning in arid and desert regions like Yazd, with a specific focus on improving human thermal comfort.

## 2. Methodology

### 2.1. Study area

The present study was conducted in the historic city of Yazd, located in central Iran amidst the desert. Yazd was selected as the focal point for an in-depth case study due to its unique climatic and geographic conditions. Positioned at a latitude of 31°54′ north, longitude 54°17′ east, and an elevation of 1,237 meters above sea level, the city receives an annual precipitation of only 71 millimeters. It experiences maximum temperatures of up to 45°C, an average annual temperature of 19°C, and an average humidity of 30% [41,42]. Situated within the equatorial belt, the climate of Yazd Province is characterized by hot, prolonged summers and arid conditions, earning Yazd its distinction as the “capital of the Iranian desert” [43]. In 2017, the Historic City of Yazd was inscribed as a UNESCO World Heritage Site under Criteria (iii) and (v), encompassing a buffer zone of 665.93 hectares [44]. This designation highlights its importance as one of the most vibrant and well-preserved traditional earthen cities in the world. Distinguished by its iconic architectural elements—including wind catchers, courtyards, Qanats, and thick earthen walls—Yazd exemplifies innovative microclimate adaptations and sustainable living practices developed over centuries in a harsh desert environment [45].

The residential area selected for this study was confined to an urban region within Zone 2 of Yazd, covering approximately 5,000 square meters. This zone represents a contemporary urban landscape predominantly developed after the 1990s. Due to the limitations of traditional earthen architecture in accommodating roof gardens, this area was deemed suitable for modeling. The selected zone primarily consists of new residential buildings constructed after the 2000s.

**Fig 1:**
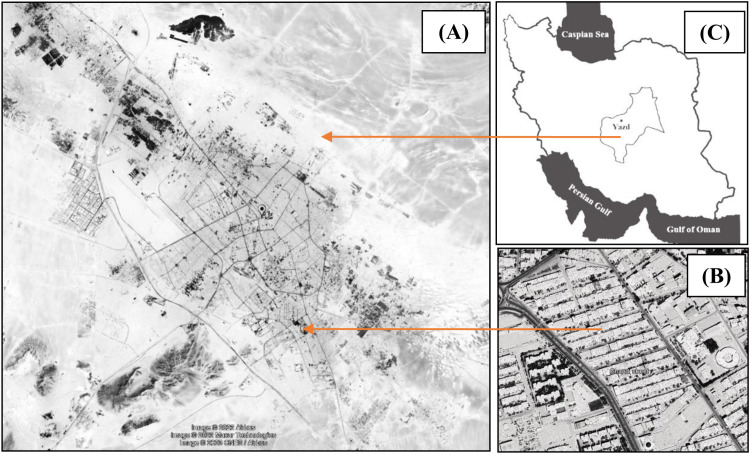
Location of simulated site: Gandhi Street (B) in the southeast of Yazd city (A), Iran (C).

Apart from the historical core of Yazd, which is located in the city center, most residential buildings in Yazd were built post-2000. The selection criteria included consistent architectural characteristics, such as construction year, building height and number of stories (two-story buildings), building materials, and orientation relative to the sun’s path. A representative sample of roofs within this residential area was surveyed, as illustrated in Fig 1, enabling a detailed exploration of urban landscape management practices and potential planning strategies in a modern urban context.

### 2.2. ENVI-met simulation

To simulate microclimate impacts and assess human thermal comfort on both extensive and intensive green roofs, ENVI-met version 3 software was utilized. ENVI-met is a three-dimensional (3D) computational fluid dynamics (CFD) model designed to perform non-hydrostatic simulations of outdoor microclimates, with broad applications in urban climate studies, architectural assessments, and environmental and building design. The ENVI-met model is based on the incompressible Reynolds-averaged Navier–Stokes (RANS) equations with the Boussinesq approximation [46,47].

This advanced model simulates surface–plant–air interactions with a spatial resolution ranging from 0.5 to 10 meters and a temporal resolution of 10 seconds. It incorporates various editing tools and graphic visualization software for processing and presenting model outputs [48].

**Fig 2:**
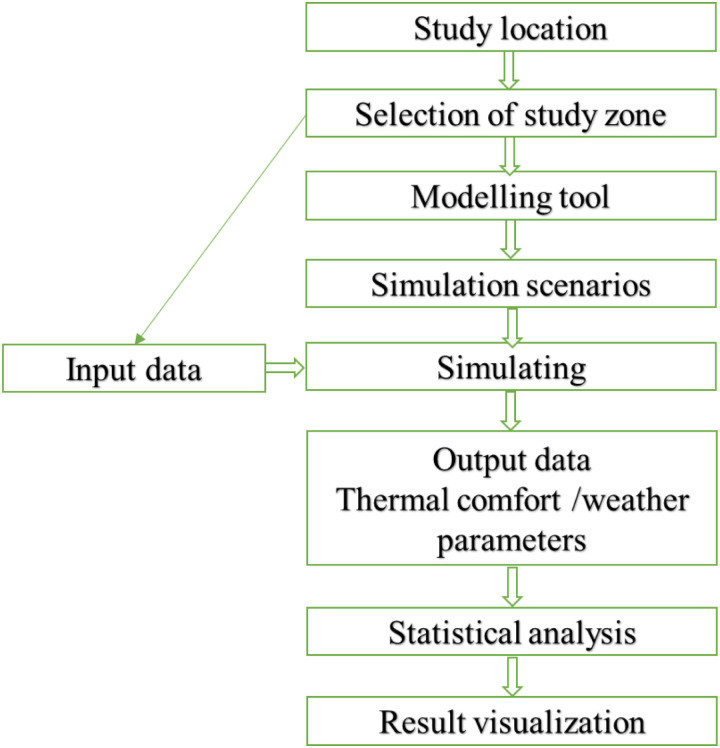
Flow diagram of the research process.

ENVI-met version 3 consists of several modules, each designed to simulate and analyze specific environmental factors in urban areas. The main modules include the microclimate simulation module, airflow simulation module, radiation simulation module, vegetation and green infrastructure simulation module, climate change impact simulation module, thermal comfort simulation module, data analysis module, and scenario simulation module [48]. In the model, plants are not only treated as permeable obstacles to wind and solar radiation but are also conceptualized as biological entities engaged in physiological processes such as transpiration, evaporation, and photosynthesis. The model supports the use of various vegetation types, each with defined characteristics. Additionally, it includes an expandable plant database, allowing users to add new plant species with specified attributes [49]. This flexibility enhances the model’s adaptability, enabling the simulation of a wide range of vegetative scenarios and making it highly effective for environmental research and urban landscape design applications. The flowchart illustrating the research process is presented in Fig 2.

### 2.3. Scenario tests

**Fig 3:**
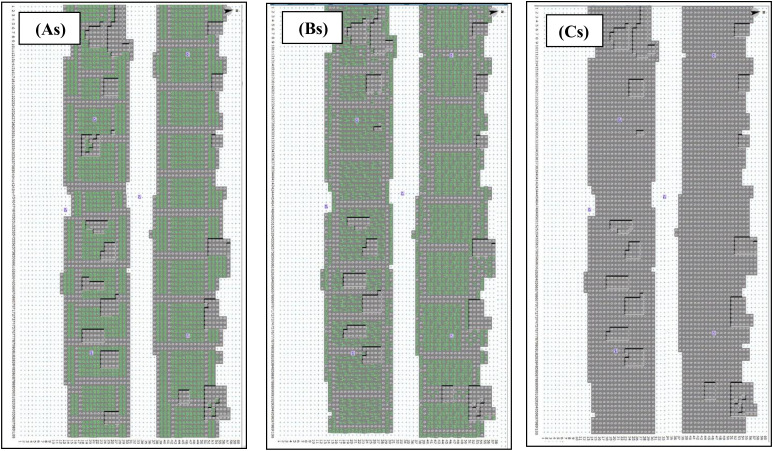
Simulated roofs: Extensive green roof (As), Intensive green roof (Bs) and Roofs without vegetation (Cs) in Yazd city/Iran, the purple points are designed receptors, Envi-met model 3.1, 2023.

Descriptions of three scenarios are provided in Tables 1 and 2, with the corresponding 3D models in ENVI-met depicted in Fig 3 to analyze the impact of different green roof types on microclimatic parameters and thermal comfort in Yazd.

**Table 1:** Scenarios tested for the studied area in Yazd, 2023.

Scenario	Code	Description
Extensive green roofs	As	80% of the roofs are covered by herbaceous and bushy plants
Intensive green roofs	Bs	80% of the roofs are covered by herbaceous plants and dense shrub hedges
Roofs without vegetation	Cs	Existing roof without any type of plants

**Table 2:** The list of plant species (adapted to arid and semi-arid areas) matching the input data in the plant database of Envi-met model.

Scientific name	Family name
*Cercis griffithii*	Fabaceae
*Cotoneaster kotschyi*	Rosaceae
Lythraceae	Lythraceae
*Punica Granatum* var. Nana	Punicaceae
*Vitex agnus-castu*	Verbenaceae
*Rosa damascene*	Rosacea
*Berberis vulgaris*	Berberidaceae
*Atriplex canescens*	Amaranthaceae
*Celosia argentea*	Amaranthaceae
*Zataria multiflora*	Lamiaceae
*Achillea millefolium*	Asteraceae
*Ephedra spp*	Ephedraceae

To simulate green roofs under industry standards and established definitions, extensive green roofs were modeled using herbaceous and bushy plants. For intensive green roofs, in addition to herbaceous vegetation, several relatively dense shrub hedges compatible with hot and dry climates were included (see Table 2).

Although specific plant species were not directly input into the ENVI-met model, its plant database includes essential characteristics that influence the microclimate, such as plant type, stomatal resistance, Leaf Area Density (LAD), root zone depth, and plant height. These parameters enable the accurate simulation of vegetation coverage. Accordingly, the selected plant species for both extensive and intensive green roofs were added to the model’s plant database for future use in subsequent simulations.

In both green roof types, approximately 80% of the roof area was simulated using the selected vegetation. In the third scenario, roofs were simulated without any vegetation cover (Fig 3). For all three scenarios, the selected roofs were set at the same height above ground level (ranging from 6 to 8 meters), and the building and roof materials were kept identical across all cases.

To obtain point-specific microclimate data from ENVI-met, receptors were selected within each simulation domain. Receptors are grid points where ENVI-met generates output data across the entire vertical profile. Six receptors were placed at the proposed site for each scenario: four on the green roofs and two within the courtyard area of the residential setting (Fig 2, marked with purple points). The placement of receptors was based on factors such as spatial dimensions, geometric configuration, and orientation. Data from the four rooftop receptors and the two courtyard receptors were averaged separately. Two representative values, one for the green roof domain and one for the courtyard domain, were then derived and used for data analysis.

### 2.4. Field measurement, data processing, and data analysis

The input data for the ENVI-met model primarily consist of four categories: meteorological data, plant data, soil data, and building data. Some of the most critical input parameters used in the model are summarized in Table 3. ENVI-met includes a built-in plant database that provides detailed information on various plant species, allowing users to model and simulate the effects of different vegetation types on urban microclimates. Plant data, based on the characteristics of species used in extensive and intensive green roof systems, were integrated into the model.

**Table 3:** Input data for ENVI-met simulation.

Input data	Measured characteristics			
Meteorological data	Air temperature	Relative humidity	Wind velocity	Wind direction
Plant data	Plant type	Plant height	LAD	Stomata resistance, …
Soil data	Soil temperature	–	–	–
Building data	Indoor temperature	U-Value Wall	U-Value Roof	–

The meteorological input parameters include air temperature, relative humidity, wind speed and direction, solar radiation, and precipitation. These data were derived from long-term weather statistics (spanning ten years) obtained from the Meteorological Organization of Yazd City and applied to a typical hot summer day in the model. The values were averaged over the hottest period of the year and used as representative input conditions. Soil temperature was calculated using established formulas and resources [50].

Building data were incorporated by considering the types and thicknesses of materials used in the buildings within the study area. The U-values for the walls (solid brick) and roofs (bituminous) were obtained from a report published by the BRE Group [51]. Simulations were conducted over a 12-hour period, from 7:00 a.m. to 6:00 p.m., during the hottest time of the year (around July) in 2023. This timeframe was selected due to the presence of sunlight, heightened human activity, and alignment with administrative working hours—all of which contribute to the highest levels of human thermal stress.

To assess thermal comfort, two thermal indices, Physiologically Equivalent Temperature (PET) and Predicted Mean Vote (PMV), available within the ENVI-met model, were utilized. These indices are widely recognized for evaluating human thermal comfort, as their calculations are based on estimating the energy balance between the human body and the surrounding environment [52]. Both indices, particularly PET, are extensively applied to assess thermal conditions in outdoor spaces, such as urban parks, streets, transportation hubs, public squares, and commercial areas, as well as in indoor settings [53,54]. Given the simulation timeframe (summer season, July), the thermal perception thresholds and physiological stress levels for both indices are presented in Table 4.

**Table 4:** Classification of the numerical threshold for thermal index: PMV in the warm period of the year [55].

PMV	Thermal perception	Grade of physiological stress
−0.5 to +05	Comfortable	No thermal stress
0.5 to 1.5	Slightly warm	Slight heat stress
1.5 to 2.5	Warm	Moderate heat stress
2.5 to 3.5	Hot	Strong heat stress
> 3.5	Very hot	Extreme heat stress

Considering that ENVI-met outputs rendered in Leonardo are presented as maps, the receptor data were exported to Excel for further analysis. The data were organized according to a factorial experimental design, encompassing the previously mentioned simulation scenarios with three receptors (x1 to x3) over a 12-hour period, each with three replications. The measured hours were grouped into four time intervals: 7:00–9:00 a.m., 10:00–12:00 p.m., 1:00–3:00 p.m., and 4:00–6:00 p.m., corresponding to the hottest period of 2023.

### 2.5. Data Analysis

Analysis of variance (ANOVA) and mean comparison for the simulated parameters were performed using Minitab 23 software, with Tukey’s test applied at a 5% significance level. Graphs were generated using Origin 2022, which was also used for data analysis and visualization. The findings from the ENVI-met model were validated by comparing the simulated data with recorded field measurements (Section 3.7).

## 3. Results

**Fig 4:**
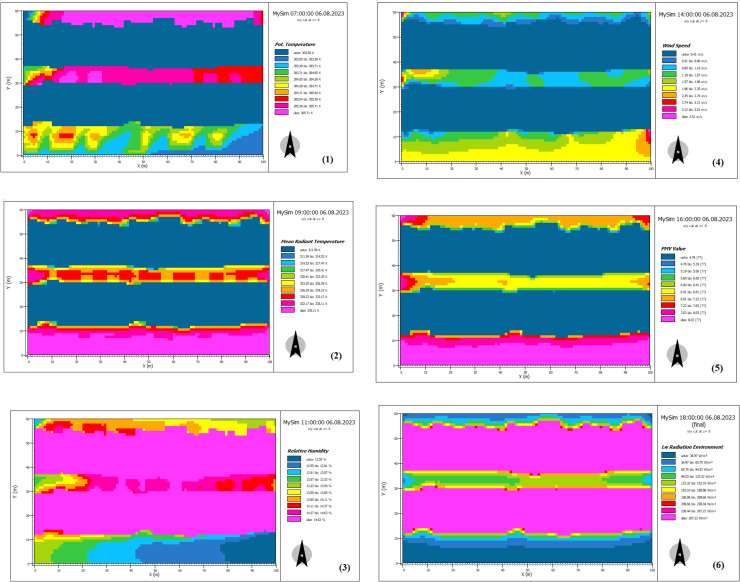
Output maps of Leonardo (ENVI-met 3.1) in some simulated scenarios and hours. (1) and (2): Extensive green roof at 7 am and 9 am respectively, (3) and (4): Intensive green roof at 11 am and 2 pm respectively, (5) and (6): Roofs without vegetation at 4 pm and 6 pm respectively.

Fig 4 presents a selection of simulated parameters, randomly chosen due to the extensive range of output maps generated in the Leonardo section (a total of 216 output maps).

As shown in Table 5, the analysis of variance (ANOVA) for the climatic parameters reveals that the main effects of scenario, receptor, and measured hours, as well as most interaction effects among these factors, were statistically significant at the 1% level. However, several exceptions were observed: the triple interaction of scenario, receptor, and hour on PMV; the main effect of hour and the interaction between scenario and hour on longwave (LW) radiation; and the interaction between scenario and receptor on mean radiant temperature (MRT) were not statistically significant (Table 5).

**Table 5:** Analysis of variance (mean square) of ENVI-met output for microclimatic parameters across three scenarios and three receptor locations over a 12-hour simulation period.

Sources	Parameter					
	Pot. Tem (°C)	RH (%)	Wind Sp. (m/s)	LW (w/m^2^)	MRT (°C)	PMV
Scenario	772.48^*^	1110.19^*^	0.0055^*^	10349^*^	473.66^*^	27.728^*^
Receptor	601.32^*^	757.67^*^	13.9471^*^	406115^*^	3170.09^*^	77.256^*^
Hour	614.24^*^	1301.66^*^	0.0670^*^	54 ^ns^	4094.51^*^	81.124^*^
Scenario×Receptor	344.36^*^	459.19^*^	0.0672^*^	6991^*^	21.29 ^ns^	7.679^*^
Scenario×Hour	35.20^*^	49.48^*^	0.0037^*^	54 ^ns^	562.44^*^	7.520^*^
Receptor×Hour	18.39^*^	23.06^*^	0.0118^*^	92^*^	276.32^*^	5.463^*^
Scenario×Receptor×Hour	8.861^*^	23.25^*^	0.0058^*^	92^*^	55.78^*^	1.072 ^ns^
Error	2.773	7.02	0.0011	41	27.62	0.448

* : Significant in 1% probability, ns: non-significant

Pot. Tem.: Potential temperature (^0^C), RH: Relative humidity (%), Wind S.: Wind speed (m/s), PMV: Predicted Mean Vote, LW: Long wave radiation environment (w/m^2^), MRT: Mean radiation temperature (^0^C)

In this section, the main effects of the simulated factors are presented first, followed by the triple interaction effects, which will be discussed in more detail in the subsequent discussion section.

### 3.1. Temperature and mean radiation temperature (MRT)

**Fig 5:**
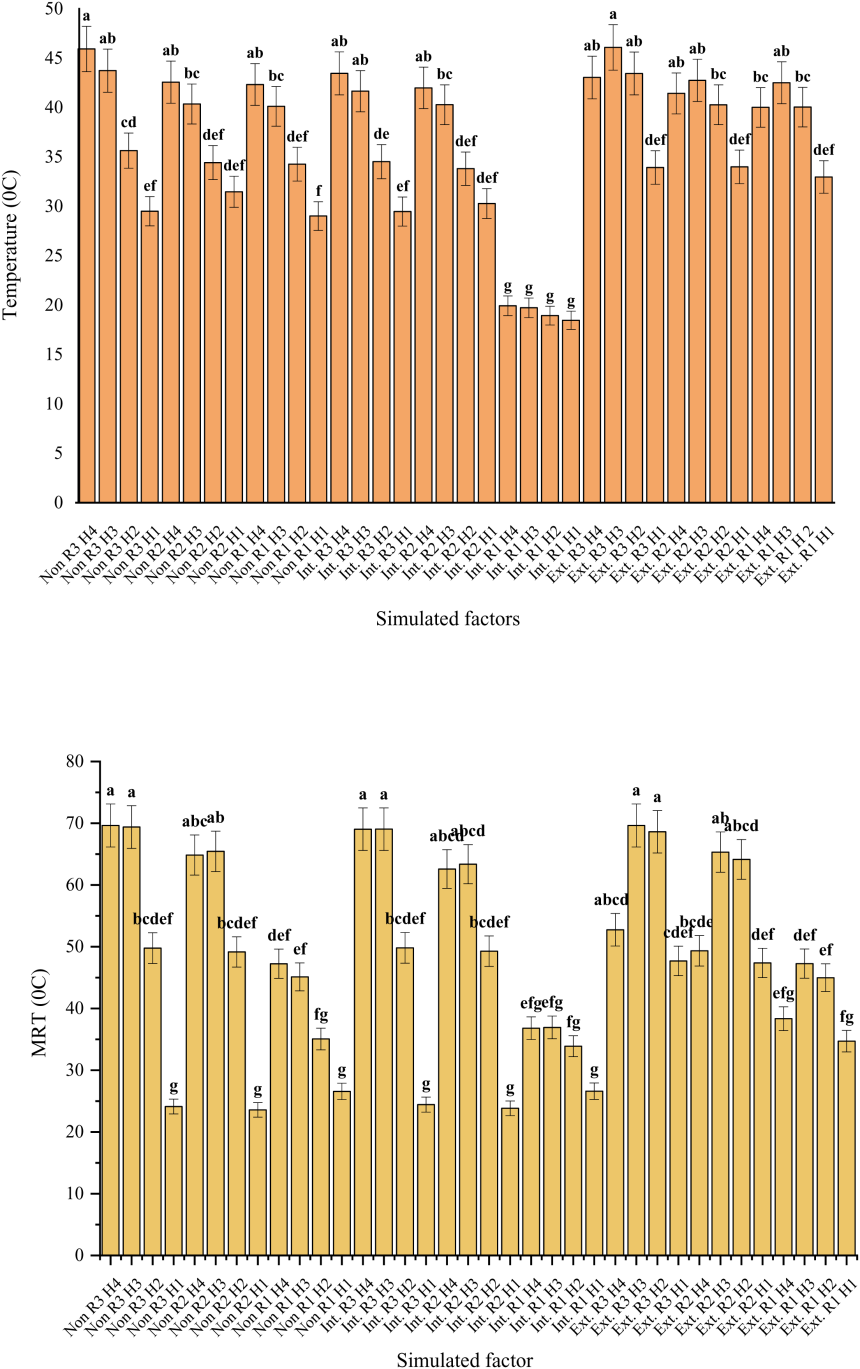
Temperature (top graph) and Mean radiation temperature (MRT) (bottom graph) of triple interactions simulated factors (roof types × receptors × measured hours) in the hottest period of the year in Yazd city, Iran.

Based on the data analysis, the highest potential temperatures were recorded in the following order: extensive green roofs (40°C), roofs without vegetation (37.5°C), and intensive green roofs (31°C). Among the receptors, the lowest potential temperatures were observed on green roofs (31.5°C), in spaces between buildings (37.8°C), and at the southern edges of buildings (39.2°C). The lowest potential temperature (29.9°C) was documented during the 7:00–9:00 a.m. period, while the highest (39.9°C) occurred between 1:00–6:00 p.m. (Fig 5). The overall lowest potential temperature (18.5°C) was simulated at the receptor located on the intensive green roof during the 7:00–9:00 a.m. period. Moreover, no significant differences were detected in the simulated potential temperature at this receptor between 7:00 a.m. and 6:00 p.m.

Conversely, the highest potential temperature (46°C) was observed on roofs without vegetation and on extensive green roofs, both located at the southern edge of buildings, during the 4:00–6:00 p.m. and 1:00–3:00 p.m. periods, respectively (Fig 5). The trends in Mean Radiant Temperature (MRT) variations across the simulated scenarios (extensive green roofs, roofs without vegetation, and intensive green roofs), receptor locations, and measured hours closely mirrored those of potential temperature. The highest MRT value (69.6°C) was recorded on roofs without vegetation and extensive green roofs at the southern edge of buildings during the 4:00–6:00 p.m. and 1:00–3:00 p.m. intervals, respectively (Fig 5).

### 3.2. Relative humidity (RH)

**Fig 6:**
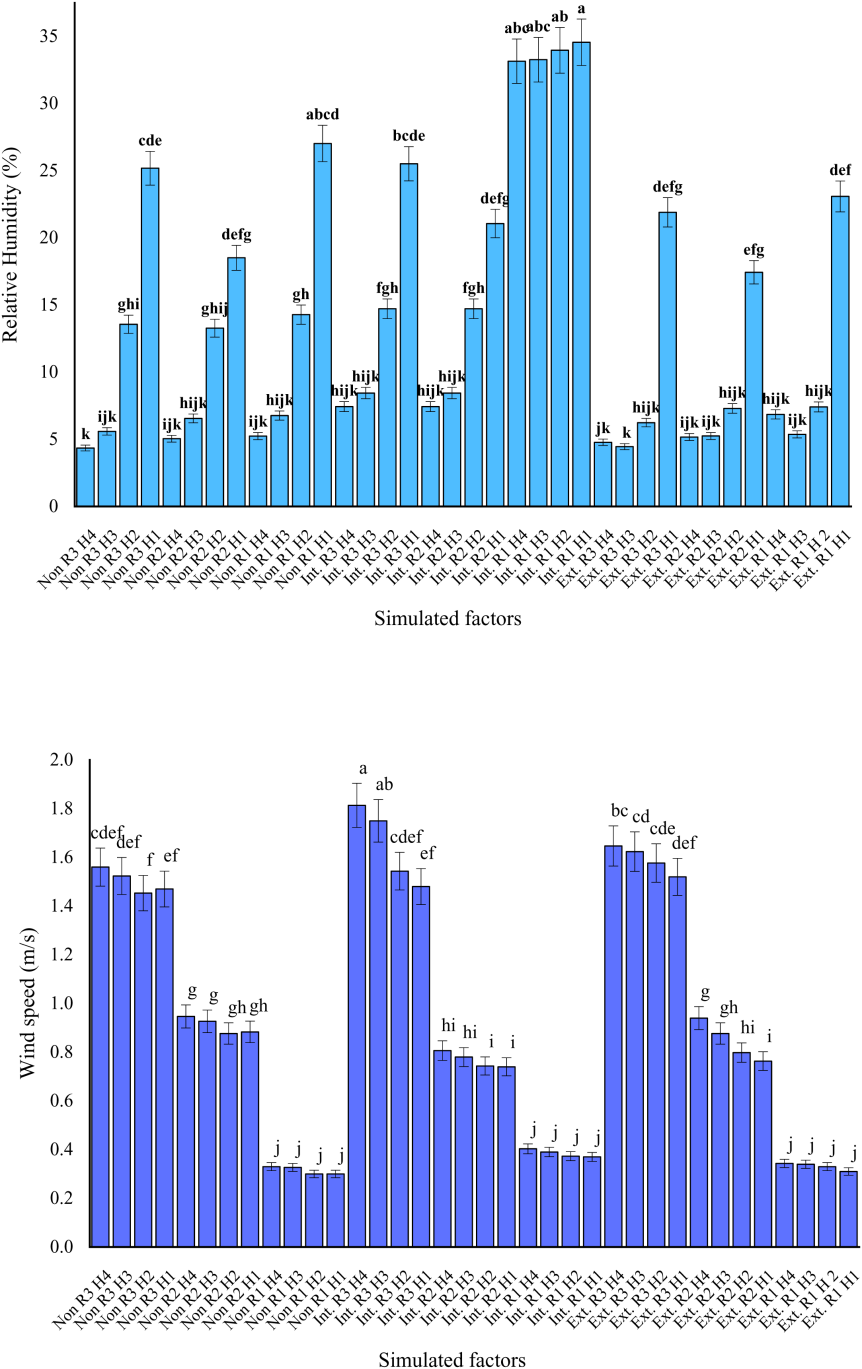
Relative humidity (top graph) and wind speed (bottom graph) of triple interactions simulated factors (roof types × receptors × measured hours) in the hottest period the year in Yazd city, Iran.

As illustrated in Fig 6, the highest Relative Humidity (RH) levels were observed in the intensive green roofs scenario (20.23%), followed by roofs without vegetation (12.12%) and extensive green roofs (9.6%). Among the receptors, the highest RH levels were recorded on green roofs (19.25%), while the lowest levels were observed in the spaces between buildings (10.85%). However, this receptor did not show a statistically significant difference in RH compared to the receptor located at the southern edges of buildings.

The highest RH value (23.81%) occurred during the 7:00–9:00 a.m. period, while the lowest (8.83%) was recorded between 4:00–6:00 p.m. Within this simulation, the highest RH level (34.57%) was documented at the receptor located on the intensive green roof during the 7:00–9:00 a.m. interval. In contrast, the lowest RH level (4.35%) was observed at the receptor on the southern edge of buildings during the 4:00–6:00 p.m. period.

### 3.3. Wind speed

Based on the data presented in Fig 6, the simulated scenarios revealed a notable difference in wind speed exclusively between the intensive green roof (0.93 m/s) and the non-vegetated roofs (0.91 m/s). Among the receptors, the highest wind speed was recorded at the southern edges of buildings (1.58 m/s), followed by the spaces between buildings (0.84 m/s), while the lowest wind speed was observed on green roofs (0.34 m/s). The highest average wind speed across all scenarios occurred during the 4:00–6:00 p.m. period (0.98 m/s), whereas the lowest (0.87 m/s) was recorded between 7:00–9:00 a.m.

Specifically, the maximum wind speed (1.81 m/s) was observed at the receptor located on the southern edge of buildings in the intensive green roof scenario during the 4:00–6:00 p.m. period. In contrast, the lowest wind speed (0.3 m/s) was simulated at the receptor on non-vegetated roofs during the 7:00–9:00 a.m. timeframe (see Fig 6).

### 3.4. Long wave (LW) Radiation

**Fig 7:**
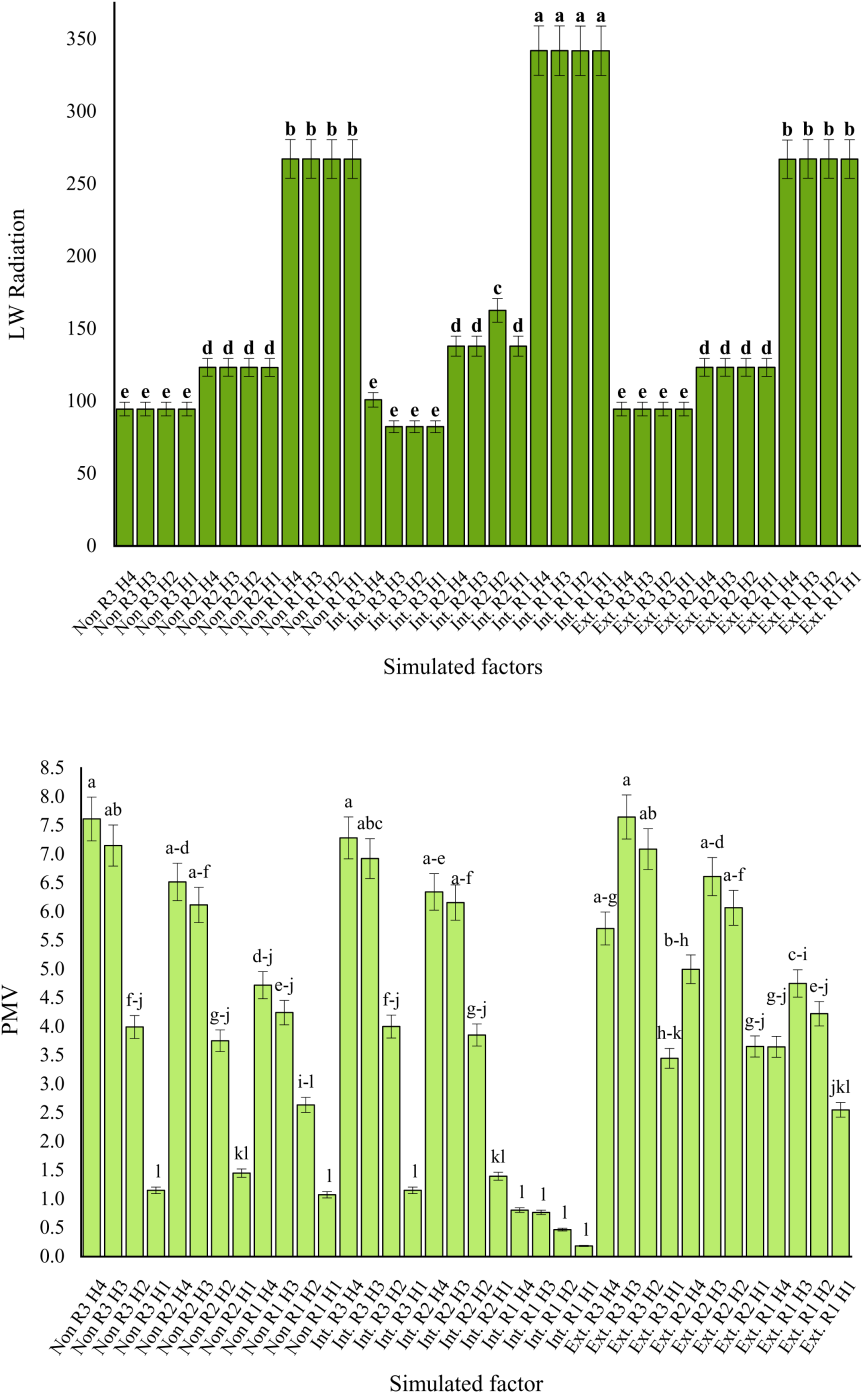
Ling wave (LW) radiation (left graph) and PMV (right graph) of triple interactions simulated factors (roof types × receptors × measured hours) in the hottest period the year in Yazd city, Iran.

The highest longwave (LW) radiation level (191 W/m²) was recorded on the intensive green roof, while the lowest level (161.6 W/m²) was observed in both the extensive green roof and non-vegetated roof scenarios, with no statistically significant difference between them (see Fig 7). Among the receptors, LW radiation levels varied as follows: the southern edges of buildings recorded the lowest value (92 W/m²), followed by the spaces between buildings (130.26 W/m²), while the highest value was observed on the roofs (292.1 W/m²). No statistically significant differences in LW radiation were detected across the simulated time intervals. However, the maximum LW radiation value (341.99 W/m²) was recorded at the receptor located on the intensive green roof during the 1:00–6:00 p.m.

### 3.5. Predicted Mean Vote (PMV)

According to Fig 7, Predicted Mean Vote (PMV) values ranged from lowest to highest across the three simulated scenarios as follows: intensive green roofs (3.2), roofs without vegetation (4.2), and extensive green roofs (5.02). Among the three receptor locations, the lowest PMV value was recorded on green roofs (2.5), followed by the spaces between buildings (4.7), with the highest value observed at the southern edges of buildings (5.3).

In addition, the lowest PMV level (1.78) was simulated during the 7:00–9:00 a.m. period, while the highest level (5.6) was recorded between 1:00–3:00 p.m. The receptor located on the intensive green roof registered the lowest PMV value (0.18) during the 7:00–9:00 a.m. timeframe. Conversely, the highest PMV levels (7.6) were recorded at the receptors positioned on the southern edges of buildings—on roofs without vegetation during 4:00–6:00 p.m., and on the extensive green roof during 1:00–3:00 p.m.

### 3.6. Changes of climatic parameters and PMV

**Fig 8:**
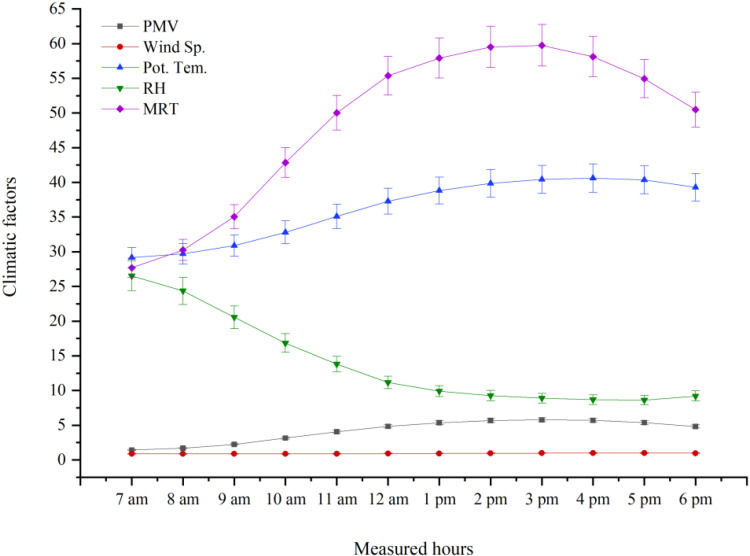
Change the trend of climatic factors and PMV from 7:00 am to 6:00 pm in the hottest period the year in Yazd city, Iran.

As shown in Fig 8, the trends for potential temperature, mean radiant temperature (MRT), and Predicted Mean Vote (PMV) followed a similar pattern, exhibiting an upward trend from 7:00 a.m. to approximately 3:00 p.m., followed by a decline until the end of the simulation at 6:00 p.m. In contrast, the trend for relative humidity (RH) was nearly inverse to these variables, although its variations remained relatively stable after 3:00 p.m. Additionally, wind speed showed a relatively constant pattern between 7:00 a.m. and 1:00 p.m., followed by a gradual increase until 6:00 p.m. It is worth noting that no statistically significant change was observed in longwave (LW) radiation levels during the simulation period; therefore, its curve was omitted from Fig 8.

### 3.7. Validation of ENVI-met output

**Fig 9:**
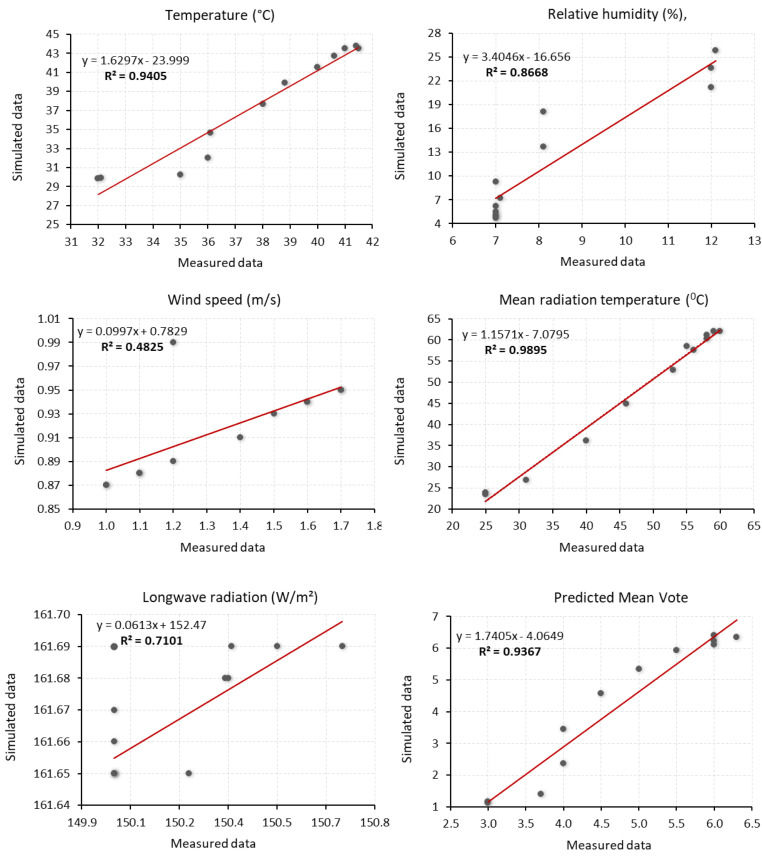
Scatter chart illustrating the coefficient of determination (R²) for measured versus simulated values: temperature (°C), relative humidity (%), wind speed (m/s), Mean radiation temperature (^0^C), longwave radiation (W/m²), and Predicted Mean Vote (PMV) during the period from 7:00 a.m. to 6:00 p.m.

The model’s simulation was validated by comparing recorded and simulated data for the period between 7:00 a.m. and 6:00 p.m. For statistical validation, three quantitative metrics were calculated: the coefficient of determination (R²), Mean Absolute Error (MAE), and Root Mean Squared Error (RMSE) [56,57]. In this study, the R² values for temperature, mean radiant temperature (MRT) and Predicted Mean Vote (PMV) were above 0.90, indicating a strong correlation between measured and simulated data. For relative humidity (RH) and longwave (LW) radiation, the R² was above 0.70, also signifying a strong correlation. In contrast, the R² value for wind speed was 0.48, indicating a moderate correlation (Fig 9 and Table 6). The MAE and RMSE values further support an acceptable level of consistency between measured and simulated data (Table 6) [56].

**Table 6:** Model validation of values: Pot. Tem, RH, Wind S, LW, MRT and PMV.

Validation Method	Pot. Tem.	RH	Wind S.	LW	MRT	PMV
Coefficient of Determination (R^2^)	0.941	0.866	0.483	0.710	0.989	0.937
Mean Absolute Error (MAE)	0.254	−1.66	0.425	−1.461	0.603	0.547
Root Mean Squared Error (RMSE)	2.492	1.852	0.486	1.463	1.767	1.145

Pot. Tem.: Potential temperature (^0^C), RH: Relative humidity (%), Wind S.: Wind speed (m/s), LW: Longwave radiation (w/m^2^), MRT: Mean radiation temperature (^0^C) and PMV: Predicted Mean Vote.

## 4. Discussion

### 4.1. Temperature and Mean radiation temperature (MRT)

As discussed in the results section, most climatic parameters examined across scenarios, receptors, and measured hours were statistically significant at the 1% level. Notably, intensive green roofs demonstrated significantly lower temperatures, by 7.75°C and 4.66°C compared to extensive green roofs and roofs without vegetation, respectively, and also exhibited reduced mean radiant temperature (MRT). This study further confirms that the integration of green roofs can effectively reduce temperatures both inside and outside buildings [14,58,59]. The benefits of vegetative cover are achieved through mechanisms such as shading, increased relative humidity, and modulation of wind speed. These mechanisms, along with key climatic factors including air temperature and solar radiation, collectively contribute to improved thermal comfort [49].

Moreover, the specific typology of green roofs has varying impacts on climatic parameters, particularly ambient temperature. Intensive green roofs, characterized by differences in plant species, vegetation density, and soil depth, demonstrate greater environmental efficacy than extensive systems. These attributes enhance their influence on the surrounding microclimate [24] and contribute to improved thermal performance [60,61]. MRT, together with air temperature, relative humidity, and wind velocity, is a critical environmental parameter within the human energy balance framework and plays a significant role in determining levels of thermal comfort [62].

Vegetative cover (plant canopy) and leaf area index (LAI) play a significant role in determining mean radiant temperature (MRT), with increased canopy density and vegetation coverage contributing to reductions in MRT [63,64]. In the present study, green roofs demonstrated lower MRT values compared to non-vegetated surfaces, primarily due to the presence of vegetative cover. However, the extensive green roofs did not show a significant reduction in air temperature or MRT when compared to roofs without vegetation. This outcome can be attributed to the lower LAI and reduced canopy density of the plant species used in the extensive green roofs, in contrast to the more densely vegetated intensive green roofs.

Similar results were reported by Khabaz (2018) in Saudi Arabia, where green roofs with shrubs outperformed those covered with turf in terms of thermal performance in hot and dry climates [65]. Likewise, a study by Perez et al. (2015) found that extensive green roofs, due to their limited vegetation and shallow substrate depth, exhibited poor temperature regulation, heating up and cooling down rapidly [66]. In the present study, receptors located on the southern sides of buildings recorded higher temperatures than those positioned between buildings or on rooftops. Vegetated areas, such as green roofs, were consistently cooler as a result of combined shading effects and evapotranspiration [49,67]. These findings are consistent with the simulation results of this experiment. In hot and dry climates, particularly during summer, MRT and air temperature were notably higher during the periods of 1:00–3:00 p.m. and 4:00–6:00 p.m., primarily due to direct solar radiation.

In arid and dry climates such as Yazd, where summer temperatures and solar radiation are extremely high and relative humidity is very low, herbaceous plants with small, narrow leaves and low shoot density may have a limited impact on microclimatic parameters. In this study, the plants used in the extensive green roofs were primarily shrubs and herbaceous native species adapted to desert conditions (Table 2).

In hot, arid, and desert regions, plant adaptations to extreme climate conditions lead to distinct morphological traits, including smaller leaf size and reduced surface area, the development of spiny stems to minimize water loss through evaporation, and the presence of waxy or oily coatings on leaf surfaces. While these adaptations enhance plant survival in harsh environments, they tend to reduce the plants’ ability to mitigate solar radiation absorption and lower surface temperatures effectively. Giagnacovo et al. (2025) reported that native species show strong potential for green roof applications. Beyond their resilience and environmental compatibility, the use of native plants is also recommended for their aesthetic value and their role in supporting urban biodiversity [68].

### 4.2. Relative humidity (RH)

The relative humidity (RH) level on intensive green roofs (20.23%) was nearly double that observed on extensive green roofs and roofs without vegetation, which had an average RH of 10.86%. Generally, green roofs increase RH levels in the surrounding environment due to the presence of vegetation. This occurs because plants enhance evapotranspiration and reduce direct surface evaporation through vegetative canopy coverage, resulting in increased ambient humidity levels [69]. As canopy size, leaf surface area, and vegetation density increase, evapotranspiration rates rise accordingly, further elevating RH levels [69–71].

Previous studies have indicated that elevated RH levels enhance the cooling effects of green roofs, thereby improving their temperature performance [15]. Additionally, green roofs can increase RH more effectively than shading from buildings or umbrellas, positively contributing to the urban thermal balance [72]. In the present study, the use of shrub plants with larger canopies and higher leaf surface indices, compared to vegetation on extensive green roofs, significantly contributed to higher RH levels. Several other studies have similarly reported elevated RH around green roofs compared to non-vegetated roofs or open spaces [69,73,74].

Higher ambient temperatures contribute to increased rates of evaporation and transpiration, thereby resulting in elevated relative humidity (RH) levels [69,75]. In the present simulation, RH levels at receptors located on green roofs were statistically higher than those measured in spaces between buildings and on the southern sides of buildings. Consistent with these findings, previous studies have indicated that vegetated areas, such as green roofs, exhibit higher RH levels due to enhanced evapotranspiration processes and reduced surface evaporation compared to adjacent areas exposed to greater sunlight [49].

The hourly trend of RH observed during the simulation was nearly inverse to that of temperature and mean radiant temperature (MRT). This phenomenon may be attributed to the effect of direct solar radiation and the subsequent rise in temperature during afternoon hours (1:00–3:00 p.m. and 4:00–6:00 p.m.), which resulted in decreased RH levels. In hot, arid regions such as Yazd, evaporation and transpiration rates significantly increase during summer months. The presence of dense vegetation, as exemplified by intensive green roofs, can effectively enhance the RH of the surrounding environment. Conversely, climatic parameters such as temperature and RH typically exhibit an inverse relationship in desert regions [76], a finding consistent with the results of this study.

### 4.3. Wind speed

Wind speed on intensive and extensive green roofs was statistically significantly higher compared to roofs without vegetation; however, this increase (approximately 0.02 m/s) was not substantial. This observation aligns with previous studies indicating that wind speed tends to increase above plant canopies [77]. Increased wind speed can enhance evapotranspiration rates in plants, subsequently raising relative humidity (RH) levels and reducing ambient temperature [78], findings that are consistent with the temperature and RH results of this study.

Wai et al. (2015), comparing buildings with and without green roofs, reported that the primary effect of green roofs was to mitigate wind fluctuations near the roof area [79]. In this research, the highest wind speeds were recorded, in descending order, at the receptors on the southern facades of buildings, in the spaces between buildings, and on the roofs. Although some studies have shown that wind speeds on rooftops are generally higher and more turbulent compared to ground-level measurements [80], the pattern and magnitude of wind flow remain highly complex and are influenced by numerous factors. For example, the geometric arrangement of buildings [81] and the type and density of vegetation [82] significantly affect wind speed and direction.

Based on this analysis, the highest wind speeds were recorded during the periods of 1:00–3:00 p.m. and 4:00–6:00 p.m. Although changes in wind speed on the roofs were found to be minor in this study, it is worth noting that peak wind speeds in arid and desert cities often occur in the afternoon due to thermal wind effects [83]. Thus, the observed increase in wind speed during these hours was anticipated.

### 4.4. Long wave (LW) Radiation

The amount of longwave (LW) radiation observed on intensive green roofs was approximately 19% higher compared to extensive green roofs and roofs without vegetation. Plants play a fundamental role in reflecting LW radiation due to their higher albedo relative to many ground surfaces, resulting in greater reflectivity [84]. LW radiation, along with variables such as temperature, relative humidity (RH), and wind speed, significantly influences thermal comfort indices, including the Predicted Mean Vote (PMV) [49].

According to the findings, the highest levels of LW radiation were recorded, in descending order, on roofs, in spaces between buildings, and on the southern facades of buildings. However, it should be noted that the relationship between building height, spatial geometry, and LW radiation is highly complex and influenced by multiple factors, including environmental conditions, city geography, and urban structures [85].

Plants influence environmental radiation and energy performance through shading, absorption, and reflection of solar energy. Green roofs also help manage solar radiation due to the reflective properties of their foliage, thus potentially improving both thermal comfort and building energy performance [74]. The denser vegetation cover found on intensive green roofs provides a larger surface area exposed to radiation, which contributes to higher LW radiation levels. Additionally, compared to lower-height surfaces, such as spaces between buildings, these roofs receive higher levels of LW radiation due to fewer obstacles blocking radiation.

### 4.5. Predicted Mean Vote (PMV) and Changes of climatic parameters

According to the results, the lowest PMV value (3.2) was observed in the intensive green roof scenario. As indicated in Table 4 and based on standard rounding rules, this value falls within the “hot” thermal perception range, corresponding to a degree of physiological stress classified as “strong heat stress.” In contrast, the PMV values for extensive green roofs and roofs without vegetation were entirely categorized within the “very hot” thermal perception range, indicating “extreme heat stress.” Studies consistent with these findings indicate that green roofs, due to their vegetation, soil layers, and drainage systems, can significantly improve thermal comfort and microclimatic conditions [23,29–32], particularly during the hottest periods of the year [13–17,26]. Additional research has also demonstrated that implementing intensive green roofs in warm-hot climates enhances the thermal environment more effectively than conventional roofs [13].

The primary variables involved in calculating the PMV thermal comfort index include air temperature, relative humidity (RH), wind speed, and radiation [27], all of which were recorded and simulated in this study. The minimum PMV values were observed between 7:00 and 9:00 a.m., while the maximum values occurred between 1:00 and 3:00 p.m. In hot and dry regions such as Yazd, implementing green roofs faces significant challenges due to harsh climatic conditions. However, under these conditions, intensive green roofs, featuring denser vegetation, deeper substrates, greater water retention capacity, and improved soil moisture following rainfall events, generally demonstrate better performance [32]. Several studies suggest that intensive green roofs, characterized by dense vegetation coverage, deeper soil layers, and efficient drainage, offer better thermal comfort than extensive green roofs [65,86].

Overall, in hot and dry climates during summer, reducing air temperature and mean radiant temperature (MRT), increasing RH, enhancing wind speed, and decreasing radiation—particularly longwave (LW) radiation, can improve thermal comfort [49]. Nevertheless, as previously discussed, consistently achieving these optimal microclimatic conditions through the implementation of green roofs, even intensive ones, is not feasible throughout all the hot hours of the year.

## 5. Conclusion

The ENVI-met model simulation of different roof types (intensive green roofs, extensive green roofs, and roofs without vegetation) in the arid city of Yazd, Iran, demonstrated that intensive green roofs had a greater moderating effect on human thermal comfort during the hottest period of the year compared to the other roof types. According to the results, although the thermal comfort level for intensive green roofs was classified as “hot,” corresponding to “strong heat stress,” their performance was still more favorable than the other two scenarios. The microclimatic and thermal comfort effects of green roofs, particularly intensive green roofs, varied based on their proximity to the installation site and the time of day. Therefore, it appears that constructing green roofs, particularly extensive types, in Yazd does not significantly improve microclimatic conditions or thermal comfort during peak daytime activity hours in the hot seasons. Although green roofs may not be recommended as an effective thermal comfort solution during these periods, Yazd’s status as a tourist destination provides opportunities for their development. By focusing on alternative functions, such as recreation and aesthetics, green roofs could enhance the appeal of historic buildings and help attract tourists, meriting further investigation. Future research is recommended to explore thermal comfort during night-time as well as during colder seasons.

## Supporting information

S1 Table Average data of recorded variables and data variance analysis. (DOCX)

S2 File Data related to the experiment. (XLSX)
