# Supplementary material for: Microclimate effects and outdoor thermal comfort of green roof types in hot and dry climates: Modelling in the historic city of Yazd, Iran
Source: PLoS One. 2025 Jun 10;20(6):e0325494. doi: 10.1371/journal.pone.0325494 (PMC12151356; doi:10.1371/journal.pone.0325494)
Supplement: S1 Table — (DOCX) [file pone.0325494.s001.docx]

WORKSHEET 1

**General Linear Model: Pot. Tempe. versus Senario, Station, Hour**

**Factor Information**

| **Factor** | **Type** | **Levels** | **Values** |
| --- | --- | --- | --- |
| Senario | Fixed | 3 | Ext., Int., non |
| Station | Fixed | 3 | 1, 2, 3 |
| Hour | Fixed | 4 | 1, 2, 3, 4 |

**Analysis of Variance**

| **Source** | **DF** | **Adj SS** | **Adj MS** | **F-Value** | **P-Value** |
| --- | --- | --- | --- | --- | --- |
| Senario | 2 | 1545.0 | 772.485 | 278.53 | 0.000 |
| Station | 2 | 1202.6 | 601.324 | 216.81 | 0.000 |
| Hour | 3 | 1842.7 | 614.246 | 221.47 | 0.000 |
| Senario*Station | 4 | 1377.5 | 344.368 | 124.17 | 0.000 |
| Senario*Hour | 6 | 211.2 | 35.203 | 12.69 | 0.000 |
| Station*Hour | 6 | 110.4 | 18.394 | 6.63 | 0.000 |
| Senario*Station*Hour | 12 | 106.3 | 8.861 | 3.20 | 0.001 |
| Error | 72 | 199.7 | 2.773 |  |  |
| Total | 107 | 6595.4 |  |  |  |

WORKSHEET 1

**General Linear Model: RH versus Senario, Station, Hour**

**Factor Information**

| **Factor** | **Type** | **Levels** | **Values** |
| --- | --- | --- | --- |
| Senario | Fixed | 3 | Ext., Int., non |
| Station | Fixed | 3 | 1, 2, 3 |
| Hour | Fixed | 4 | 1, 2, 3, 4 |

**Analysis of Variance**

| **Source** | **DF** | **Adj SS** | **Adj MS** | **F-Value** | **P-Value** |
| --- | --- | --- | --- | --- | --- |
| Senario | 2 | 2220.4 | 1110.19 | 158.16 | 0.000 |
| Station | 2 | 1515.3 | 757.67 | 107.94 | 0.000 |
| Hour | 3 | 3905.0 | 1301.66 | 185.44 | 0.000 |
| Senario*Station | 4 | 1836.7 | 459.19 | 65.42 | 0.000 |
| Senario*Hour | 6 | 296.9 | 49.48 | 7.05 | 0.000 |
| Station*Hour | 6 | 138.4 | 23.06 | 3.29 | 0.007 |
| Senario*Station*Hour | 12 | 279.0 | 23.25 | 3.31 | 0.001 |
| Error | 72 | 505.4 | 7.02 |  |  |
| Total | 107 | 10697.1 |  |  |  |

WORKSHEET 1

**General Linear Model: Wind speed versus Senario, Station, Hour**

**Factor Information**

| **Factor** | **Type** | **Levels** | **Values** |
| --- | --- | --- | --- |
| Senario | Fixed | 3 | Ext., Int., non |
| Station | Fixed | 3 | 1, 2, 3 |
| Hour | Fixed | 4 | 1, 2, 3, 4 |

**Analysis of Variance**

| **Source** | **DF** | **Adj SS** | **Adj MS** | **F-Value** | **P-Value** |
| --- | --- | --- | --- | --- | --- |
| Senario | 2 | 0.0111 | 0.0055 | 5.15 | 0.008 |
| Station | 2 | 27.8942 | 13.9471 | 12943.39 | 0.000 |
| Hour | 3 | 0.2011 | 0.0670 | 62.22 | 0.000 |
| Senario*Station | 4 | 0.2690 | 0.0672 | 62.40 | 0.000 |
| Senario*Hour | 6 | 0.0220 | 0.0037 | 3.41 | 0.005 |
| Station*Hour | 6 | 0.0708 | 0.0118 | 10.94 | 0.000 |
| Senario*Station*Hour | 12 | 0.0692 | 0.0058 | 5.35 | 0.000 |
| Error | 72 | 0.0776 | 0.0011 |  |  |
| Total | 107 | 28.6150 |  |  |  |

WORKSHEET 1

**General Linear Model: PMV versus Senario, Station, Hour**

**Factor Information**

| **Factor** | **Type** | **Levels** | **Values** |
| --- | --- | --- | --- |
| Senario | Fixed | 3 | Ext., Int., non |
| Station | Fixed | 3 | 1, 2, 3 |
| Hour | Fixed | 4 | 1, 2, 3, 4 |

**Analysis of Variance**

| **Source** | **DF** | **Adj SS** | **Adj MS** | **F-Value** | **P-Value** |
| --- | --- | --- | --- | --- | --- |
| Senario | 2 | 55.46 | 27.7288 | 61.80 | 0.000 |
| Station | 2 | 154.51 | 77.2561 | 172.19 | 0.000 |
| Hour | 3 | 243.37 | 81.1246 | 180.81 | 0.000 |
| Senario*Station | 4 | 30.72 | 7.6791 | 17.11 | 0.000 |
| Senario*Hour | 6 | 45.12 | 7.5200 | 16.76 | 0.000 |
| Station*Hour | 6 | 32.78 | 5.4630 | 12.18 | 0.000 |
| Senario*Station*Hour | 12 | 12.87 | 1.0728 | 2.39 | 0.012 |
| Error | 72 | 32.31 | 0.4487 |  |  |
| Total | 107 | 607.14 |  |  |  |

WORKSHEET 1

**General Linear Model: LW versus Senario, Station, Hour**

**Factor Information**

| **Factor** | **Type** | **Levels** | **Values** |
| --- | --- | --- | --- |
| Senario | Fixed | 3 | Ext., Int., non |
| Station | Fixed | 3 | 1, 2, 3 |
| Hour | Fixed | 4 | 1, 2, 3, 4 |

**Analysis of Variance**

| **Source** | **DF** | **Adj SS** | **Adj MS** | **F-Value** | **P-Value** |
| --- | --- | --- | --- | --- | --- |
| Senario | 2 | 20699 | 10349 | 249.99 | 0.000 |
| Station | 2 | 812230 | 406115 | 9809.58 | 0.000 |
| Hour | 3 | 162 | 54 | 1.30 | 0.280 |
| Senario*Station | 4 | 27964 | 6991 | 168.86 | 0.000 |
| Senario*Hour | 6 | 326 | 54 | 1.31 | 0.263 |
| Station*Hour | 6 | 555 | 92 | 2.23 | 0.049 |
| Senario*Station*Hour | 12 | 1107 | 92 | 2.23 | 0.019 |
| Error | 72 | 2981 | 41 |  |  |
| Total | 107 | 866023 |  |  |  |

WORKSHEET 1

**General Linear Model: MRT versus Senario, Station, Hour**

**Factor Information**

| **Factor** | **Type** | **Levels** | **Values** |
| --- | --- | --- | --- |
| Senario | Fixed | 3 | Ext., Int., non |
| Station | Fixed | 3 | 1, 2, 3 |
| Hour | Fixed | 4 | 1, 2, 3, 4 |

**Analysis of Variance**

| **Source** | **DF** | **Adj SS** | **Adj MS** | **F-Value** | **P-Value** |
| --- | --- | --- | --- | --- | --- |
| Senario | 2 | 947.3 | 473.66 | 17.15 | 0.000 |
| Station | 2 | 6340.2 | 3170.09 | 114.76 | 0.000 |
| Hour | 3 | 12283.5 | 4094.51 | 148.23 | 0.000 |
| Senario*Station | 4 | 85.2 | 21.29 | 0.77 | 0.548 |
| Senario*Hour | 6 | 3374.7 | 562.44 | 20.36 | 0.000 |
| Station*Hour | 6 | 1657.9 | 276.32 | 10.00 | 0.000 |
| Senario*Station*Hour | 12 | 669.3 | 55.78 | 2.02 | 0.034 |
| Error | 72 | 1988.8 | 27.62 |  |  |
| Total | 107 | 27347.0 |  |  |  |

WORKSHEET 1

**Comparisons for Pot. Tempe.**

**Tukey Pairwise Comparisons: Senario**

**Grouping Information Using the Tukey Method and 95% Confidence**

| **Senario** | **N** | **Mean** | **Grouping** | | |
| --- | --- | --- | --- | --- | --- |
| Ext. | 36 | 313.053 | A |  |  |
| non | 36 | 310.456 |  | B |  |
| Int. | 36 | 304.053 |  |  | C |

*Means that do not share a letter are significantly different.*

**Tukey Pairwise Comparisons: Station**

**Grouping Information Using the Tukey Method and 95% Confidence**

| **Station** | **N** | **Mean** | **Grouping** | | |
| --- | --- | --- | --- | --- | --- |
| 3 | 36 | 312.212 | A |  |  |
| 2 | 36 | 310.812 |  | B |  |
| 1 | 36 | 304.538 |  |  | C |

*Means that do not share a letter are significantly different.*

**Tukey Pairwise Comparisons: Hour**

**Grouping Information Using the Tukey Method and 95% Confidence**

| **Hour** | **N** | **Mean** | **Grouping** | | |
| --- | --- | --- | --- | --- | --- |
| 4 | 27 | 313.087 | A |  |  |
| 3 | 27 | 312.702 | A |  |  |
| 2 | 27 | 308.050 |  | B |  |
| 1 | 27 | 302.910 |  |  | C |

*Means that do not share a letter are significantly different.*

**Tukey Pairwise Comparisons: Senario*Station**

**Grouping Information Using the Tukey Method and 95% Confidence**

| **Senario*Station** | **N** | **Mean** | **Grouping** | | | | | |
| --- | --- | --- | --- | --- | --- | --- | --- | --- |
| Ext. 3 | 12 | 314.639 | A |  |  |  |  |  |
| Ext. 2 | 12 | 312.623 | A | B |  |  |  |  |
| Ext. 1 | 12 | 311.896 |  | B | C |  |  |  |
| non 3 | 12 | 311.711 |  | B | C | D |  |  |
| Int. 3 | 12 | 310.286 |  |  | C | D | E |  |
| non 2 | 12 | 310.216 |  |  | C | D | E |  |
| Int. 2 | 12 | 309.597 |  |  |  | D | E |  |
| non 1 | 12 | 309.442 |  |  |  |  | E |  |
| Int. 1 | 12 | 292.275 |  |  |  |  |  | F |

*Means that do not share a letter are significantly different.*

**Tukey Pairwise Comparisons: Senario*Hour**

**Grouping Information Using the Tukey Method and 95% Confidence**

| **Senario*Hour** | **N** | **Mean** | **Grouping** | | | |
| --- | --- | --- | --- | --- | --- | --- |
| Ext. 3 | 9 | 316.792 | A |  |  |  |
| non 4 | 9 | 316.619 | A |  |  |  |
| Ext. 4 | 9 | 314.507 | A |  |  |  |
| non 3 | 9 | 314.413 | A |  |  |  |
| Ext. 2 | 9 | 314.268 | A |  |  |  |
| Int. 4 | 9 | 308.134 |  | B |  |  |
| non 2 | 9 | 307.786 |  | B |  |  |
| Int. 3 | 9 | 306.900 |  | B |  |  |
| Ext. 1 | 9 | 306.644 |  | B |  |  |
| non 1 | 9 | 303.008 |  |  | C |  |
| Int. 2 | 9 | 302.098 |  |  | C |  |
| Int. 1 | 9 | 299.079 |  |  |  | D |

*Means that do not share a letter are significantly different.*

**Tukey Pairwise Comparisons: Station*Hour**

**Grouping Information Using the Tukey Method and 95% Confidence**

| **Station*Hour** | **N** | **Mean** | **Grouping** | | | | | | |
| --- | --- | --- | --- | --- | --- | --- | --- | --- | --- |
| 3 4 | 9 | 317.153 | A |  |  |  |  |  |  |
| 3 3 | 9 | 316.837 | A |  |  |  |  |  |  |
| 2 4 | 9 | 315.004 | A | B |  |  |  |  |  |
| 2 3 | 9 | 314.140 |  | B |  |  |  |  |  |
| 3 2 | 9 | 310.878 |  |  | C |  |  |  |  |
| 2 2 | 9 | 309.180 |  |  | C | D |  |  |  |
| 1 3 | 9 | 307.129 |  |  |  | D | E |  |  |
| 1 4 | 9 | 307.102 |  |  |  | D | E |  |  |
| 2 1 | 9 | 304.924 |  |  |  |  | E | F |  |
| 1 2 | 9 | 304.093 |  |  |  |  |  | F |  |
| 3 1 | 9 | 303.980 |  |  |  |  |  | F |  |
| 1 1 | 9 | 299.827 |  |  |  |  |  |  | G |

*Means that do not share a letter are significantly different.*

**Tukey Pairwise Comparisons: Senario*Station*Hour**

**Grouping Information Using the Tukey Method and 95% Confidence**

| **Senario*Station*Hour** | **N** | **Mean** | **Grouping** | | | | | | |
| --- | --- | --- | --- | --- | --- | --- | --- | --- | --- |
| Ext. 3 3 | 3 | 319.100 | A |  |  |  |  |  |  |
| non 3 4 | 3 | 318.940 | A |  |  |  |  |  |  |
| non 3 3 | 3 | 316.743 | A | B |  |  |  |  |  |
| Int. 3 4 | 3 | 316.467 | A | B |  |  |  |  |  |
| Ext. 3 2 | 3 | 316.460 | A | B |  |  |  |  |  |
| Ext. 3 4 | 3 | 316.053 | A | B |  |  |  |  |  |
| Ext. 2 3 | 3 | 315.757 | A | B |  |  |  |  |  |
| non 2 4 | 3 | 315.577 | A | B |  |  |  |  |  |
| Ext. 1 3 | 3 | 315.520 | A | B |  |  |  |  |  |
| non 1 4 | 3 | 315.340 | A | B |  |  |  |  |  |
| Int. 2 4 | 3 | 314.997 | A | B |  |  |  |  |  |
| Int. 3 3 | 3 | 314.667 | A | B |  |  |  |  |  |
| Ext. 2 4 | 3 | 314.440 | A | B |  |  |  |  |  |
| non 2 3 | 3 | 313.370 |  | B | C |  |  |  |  |
| Int. 2 3 | 3 | 313.293 |  | B | C |  |  |  |  |
| Ext. 2 2 | 3 | 313.287 |  | B | C |  |  |  |  |
| non 1 3 | 3 | 313.127 |  | B | C |  |  |  |  |
| Ext. 1 2 | 3 | 313.057 |  | B | C |  |  |  |  |
| Ext. 1 4 | 3 | 313.027 |  | B | C |  |  |  |  |
| non 3 2 | 3 | 308.643 |  |  | C | D |  |  |  |
| Int. 3 2 | 3 | 307.530 |  |  |  | D | E |  |  |
| non 2 2 | 3 | 307.437 |  |  |  | D | E | F |  |
| non 1 2 | 3 | 307.277 |  |  |  | D | E | F |  |
| Ext. 2 1 | 3 | 307.010 |  |  |  | D | E | F |  |
| Ext. 3 1 | 3 | 306.943 |  |  |  | D | E | F |  |
| Int. 2 2 | 3 | 306.817 |  |  |  | D | E | F |  |
| Ext. 1 1 | 3 | 305.980 |  |  |  | D | E | F |  |
| non 2 1 | 3 | 304.480 |  |  |  | D | E | F |  |
| Int. 2 1 | 3 | 303.283 |  |  |  | D | E | F |  |
| non 3 1 | 3 | 302.517 |  |  |  |  | E | F |  |
| Int. 3 1 | 3 | 302.480 |  |  |  |  | E | F |  |
| non 1 1 | 3 | 302.027 |  |  |  |  |  | F |  |
| Int. 1 4 | 3 | 292.940 |  |  |  |  |  |  | G |
| Int. 1 3 | 3 | 292.740 |  |  |  |  |  |  | G |
| Int. 1 2 | 3 | 291.947 |  |  |  |  |  |  | G |
| Int. 1 1 | 3 | 291.473 |  |  |  |  |  |  | G |

*Means that do not share a letter are significantly different.*

WORKSHEET 1

**Comparisons for RH**

**Tukey Pairwise Comparisons: Senario**

**Grouping Information Using the Tukey Method and 95% Confidence**

| **Senario** | **N** | **Mean** | **Grouping** | | |
| --- | --- | --- | --- | --- | --- |
| Int. | 36 | 20.2275 | A |  |  |
| non | 36 | 12.1156 |  | B |  |
| Ext. | 36 | 9.6017 |  |  | C |

*Means that do not share a letter are significantly different.*

**Tukey Pairwise Comparisons: Station**

**Grouping Information Using the Tukey Method and 95% Confidence**

| **Station** | **N** | **Mean** | **Grouping** | |
| --- | --- | --- | --- | --- |
| 1 | 36 | 19.2475 | A |  |
| 3 | 36 | 11.8475 |  | B |
| 2 | 36 | 10.8497 |  | B |

*Means that do not share a letter are significantly different.*

**Tukey Pairwise Comparisons: Hour**

**Grouping Information Using the Tukey Method and 95% Confidence**

| **Hour** | **N** | **Mean** | **Grouping** | | |
| --- | --- | --- | --- | --- | --- |
| 1 | 27 | 23.8096 | A |  |  |
| 2 | 27 | 13.9415 |  | B |  |
| 3 | 27 | 9.3474 |  |  | C |
| 4 | 27 | 8.8278 |  |  | C |

*Means that do not share a letter are significantly different.*

**Tukey Pairwise Comparisons: Senario*Station**

**Grouping Information Using the Tukey Method and 95% Confidence**

| **Senario*Station** | **N** | **Mean** | **Grouping** | | |
| --- | --- | --- | --- | --- | --- |
| Int. 1 | 12 | 33.7367 | A |  |  |
| Int. 3 | 12 | 14.0292 |  | B |  |
| non 1 | 12 | 13.3275 |  | B |  |
| Int. 2 | 12 | 12.9167 |  | B |  |
| non 3 | 12 | 12.1725 |  | B | C |
| non 2 | 12 | 10.8467 |  | B | C |
| Ext. 1 | 12 | 10.6783 |  | B | C |
| Ext. 3 | 12 | 9.3408 |  |  | C |
| Ext. 2 | 12 | 8.7858 |  |  | C |

*Means that do not share a letter are significantly different.*

**Tukey Pairwise Comparisons: Senario*Hour**

**Grouping Information Using the Tukey Method and 95% Confidence**

| **Senario*Hour** | **N** | **Mean** | **Grouping** | | | | |
| --- | --- | --- | --- | --- | --- | --- | --- |
| Int. 1 | 9 | 27.0500 | A |  |  |  |  |
| non 1 | 9 | 23.5733 | A | B |  |  |  |
| Int. 2 | 9 | 21.1344 |  | B |  |  |  |
| Ext. 1 | 9 | 20.8056 |  | B | C |  |  |
| Int. 3 | 9 | 16.7189 |  |  | C | D |  |
| Int. 4 | 9 | 16.0067 |  |  |  | D |  |
| non 2 | 9 | 13.7078 |  |  |  | D |  |
| Ext. 2 | 9 | 6.9822 |  |  |  |  | E |
| non 3 | 9 | 6.3022 |  |  |  |  | E |
| Ext. 4 | 9 | 5.5978 |  |  |  |  | E |
| Ext. 3 | 9 | 5.0211 |  |  |  |  | E |
| non 4 | 9 | 4.8789 |  |  |  |  | E |

*Means that do not share a letter are significantly different.*

**Tukey Pairwise Comparisons: Station*Hour**

**Grouping Information Using the Tukey Method and 95% Confidence**

| **Station*Hour** | **N** | **Mean** | **Grouping** | | | |
| --- | --- | --- | --- | --- | --- | --- |
| 1 1 | 9 | 28.2244 | A |  |  |  |
| 3 1 | 9 | 24.2000 | A |  |  |  |
| 2 1 | 9 | 19.0044 |  | B |  |  |
| 1 2 | 9 | 18.5522 |  | B |  |  |
| 1 3 | 9 | 15.1311 |  | B | C |  |
| 1 4 | 9 | 15.0822 |  | B | C |  |
| 2 2 | 9 | 11.7644 |  |  | C |  |
| 3 2 | 9 | 11.5078 |  |  | C |  |
| 2 3 | 9 | 6.7489 |  |  |  | D |
| 3 3 | 9 | 6.1622 |  |  |  | D |
| 2 4 | 9 | 5.8811 |  |  |  | D |
| 3 4 | 9 | 5.5200 |  |  |  | D |

*Means that do not share a letter are significantly different.*

**Tukey Pairwise Comparisons: Senario*Station*Hour**

**Grouping Information Using the Tukey Method and 95% Confidence**

| **Senario*Station*Hour** | **N** | **Mean** | **Grouping** | | | | | | | | | | |
| --- | --- | --- | --- | --- | --- | --- | --- | --- | --- | --- | --- | --- | --- |
| Int. 1 1 | 3 | 34.5667 | A |  |  |  |  |  |  |  |  |  |  |
| Int. 1 2 | 3 | 33.9633 | A | B |  |  |  |  |  |  |  |  |  |
| Int. 1 3 | 3 | 33.2633 | A | B | C |  |  |  |  |  |  |  |  |
| Int. 1 4 | 3 | 33.1533 | A | B | C |  |  |  |  |  |  |  |  |
| non 1 1 | 3 | 27.0233 | A | B | C | D |  |  |  |  |  |  |  |
| Int. 3 1 | 3 | 25.5167 |  | B | C | D | E |  |  |  |  |  |  |
| non 3 1 | 3 | 25.1833 |  |  | C | D | E |  |  |  |  |  |  |
| Ext. 1 1 | 3 | 23.0833 |  |  |  | D | E | F |  |  |  |  |  |
| Ext. 3 1 | 3 | 21.9000 |  |  |  | D | E | F | G |  |  |  |  |
| Int. 2 1 | 3 | 21.0667 |  |  |  | D | E | F | G |  |  |  |  |
| non 2 1 | 3 | 18.5133 |  |  |  | D | E | F | G |  |  |  |  |
| Ext. 2 1 | 3 | 17.4333 |  |  |  |  | E | F | G |  |  |  |  |
| Int. 2 2 | 3 | 14.7200 |  |  |  |  |  | F | G | H |  |  |  |
| Int. 3 2 | 3 | 14.7200 |  |  |  |  |  | F | G | H |  |  |  |
| non 1 2 | 3 | 14.2833 |  |  |  |  |  |  | G | H |  |  |  |
| non 3 2 | 3 | 13.5633 |  |  |  |  |  |  | G | H | I |  |  |
| non 2 2 | 3 | 13.2767 |  |  |  |  |  |  | G | H | I | J |  |
| Int. 2 3 | 3 | 8.4467 |  |  |  |  |  |  |  | H | I | J | K |
| Int. 3 3 | 3 | 8.4467 |  |  |  |  |  |  |  | H | I | J | K |
| Int. 2 4 | 3 | 7.4333 |  |  |  |  |  |  |  | H | I | J | K |
| Int. 3 4 | 3 | 7.4333 |  |  |  |  |  |  |  | H | I | J | K |
| Ext. 1 2 | 3 | 7.4100 |  |  |  |  |  |  |  | H | I | J | K |
| Ext. 2 2 | 3 | 7.2967 |  |  |  |  |  |  |  | H | I | J | K |
| Ext. 1 4 | 3 | 6.8533 |  |  |  |  |  |  |  | H | I | J | K |
| non 1 3 | 3 | 6.7633 |  |  |  |  |  |  |  | H | I | J | K |
| non 2 3 | 3 | 6.5533 |  |  |  |  |  |  |  | H | I | J | K |
| Ext. 3 2 | 3 | 6.2400 |  |  |  |  |  |  |  | H | I | J | K |
| non 3 3 | 3 | 5.5900 |  |  |  |  |  |  |  |  | I | J | K |
| Ext. 1 3 | 3 | 5.3667 |  |  |  |  |  |  |  |  | I | J | K |
| Ext. 2 3 | 3 | 5.2467 |  |  |  |  |  |  |  |  | I | J | K |
| non 1 4 | 3 | 5.2400 |  |  |  |  |  |  |  |  | I | J | K |
| Ext. 2 4 | 3 | 5.1667 |  |  |  |  |  |  |  |  | I | J | K |
| non 2 4 | 3 | 5.0433 |  |  |  |  |  |  |  |  | I | J | K |
| Ext. 3 4 | 3 | 4.7733 |  |  |  |  |  |  |  |  |  | J | K |
| Ext. 3 3 | 3 | 4.4500 |  |  |  |  |  |  |  |  |  |  | K |
| non 3 4 | 3 | 4.3533 |  |  |  |  |  |  |  |  |  |  | K |

*Means that do not share a letter are significantly different.*

WORKSHEET 1

**Comparisons for Wind speed**

**Tukey Pairwise Comparisons: Senario**

**Grouping Information Using the Tukey Method and 95% Confidence**

| **Senario** | **N** | **Mean** | **Grouping** | |
| --- | --- | --- | --- | --- |
| Int. | 36 | 0.932778 | A |  |
| Ext. | 36 | 0.922361 | A | B |
| non | 36 | 0.908056 |  | B |

*Means that do not share a letter are significantly different.*

**Tukey Pairwise Comparisons: Station**

**Grouping Information Using the Tukey Method and 95% Confidence**

| **Station** | **N** | **Mean** | **Grouping** | | |
| --- | --- | --- | --- | --- | --- |
| 3 | 36 | 1.58000 | A |  |  |
| 2 | 36 | 0.84014 |  | B |  |
| 1 | 36 | 0.34306 |  |  | C |

*Means that do not share a letter are significantly different.*

**Tukey Pairwise Comparisons: Hour**

**Grouping Information Using the Tukey Method and 95% Confidence**

| **Hour** | **N** | **Mean** | **Grouping** | | |
| --- | --- | --- | --- | --- | --- |
| 4 | 27 | 0.976667 | A |  |  |
| 3 | 27 | 0.948519 |  | B |  |
| 2 | 27 | 0.888333 |  |  | C |
| 1 | 27 | 0.870741 |  |  | C |

*Means that do not share a letter are significantly different.*

**Tukey Pairwise Comparisons: Senario*Station**

**Grouping Information Using the Tukey Method and 95% Confidence**

| **Senario*Station** | **N** | **Mean** | **Grouping** | | | | | | | |
| --- | --- | --- | --- | --- | --- | --- | --- | --- | --- | --- |
| Int. 3 | 12 | 1.64667 | A |  |  |  |  |  |  |  |
| Ext. 3 | 12 | 1.59167 |  | B |  |  |  |  |  |  |
| non 3 | 12 | 1.50167 |  |  | C |  |  |  |  |  |
| non 2 | 12 | 0.90833 |  |  |  | D |  |  |  |  |
| Ext. 2 | 12 | 0.84458 |  |  |  |  | E |  |  |  |
| Int. 2 | 12 | 0.76750 |  |  |  |  |  | F |  |  |
| Int. 1 | 12 | 0.38417 |  |  |  |  |  |  | G |  |
| Ext. 1 | 12 | 0.33083 |  |  |  |  |  |  |  | H |
| non 1 | 12 | 0.31417 |  |  |  |  |  |  |  | H |

*Means that do not share a letter are significantly different.*

**Tukey Pairwise Comparisons: Senario*Hour**

**Grouping Information Using the Tukey Method and 95% Confidence**

| **Senario*Hour** | **N** | **Mean** | **Grouping** | | | | |
| --- | --- | --- | --- | --- | --- | --- | --- |
| Int. 4 | 9 | 1.00778 | A |  |  |  |  |
| Ext. 4 | 9 | 0.97667 | A | B |  |  |  |
| Int. 3 | 9 | 0.97333 | A | B |  |  |  |
| Ext. 3 | 9 | 0.94667 |  | B | C |  |  |
| non 4 | 9 | 0.94556 |  | B | C |  |  |
| non 3 | 9 | 0.92556 |  | B | C | D |  |
| Ext. 2 | 9 | 0.90167 |  |  | C | D | E |
| Int. 2 | 9 | 0.88667 |  |  |  | D | E |
| non 1 | 9 | 0.88444 |  |  |  | D | E |
| non 2 | 9 | 0.87667 |  |  |  | D | E |
| Ext. 1 | 9 | 0.86444 |  |  |  |  | E |
| Int. 1 | 9 | 0.86333 |  |  |  |  | E |

*Means that do not share a letter are significantly different.*

**Tukey Pairwise Comparisons: Station*Hour**

**Grouping Information Using the Tukey Method and 95% Confidence**

| **Station*Hour** | **N** | **Mean** | **Grouping** | | | | |
| --- | --- | --- | --- | --- | --- | --- | --- |
| 3 4 | 9 | 1.67333 | A |  |  |  |  |
| 3 3 | 9 | 1.63222 | A |  |  |  |  |
| 3 2 | 9 | 1.52444 |  | B |  |  |  |
| 3 1 | 9 | 1.49000 |  | B |  |  |  |
| 2 4 | 9 | 0.89778 |  |  | C |  |  |
| 2 3 | 9 | 0.86111 |  |  | C |  |  |
| 2 2 | 9 | 0.80611 |  |  |  | D |  |
| 2 1 | 9 | 0.79556 |  |  |  | D |  |
| 1 4 | 9 | 0.35889 |  |  |  |  | E |
| 1 3 | 9 | 0.35222 |  |  |  |  | E |
| 1 2 | 9 | 0.33444 |  |  |  |  | E |
| 1 1 | 9 | 0.32667 |  |  |  |  | E |

*Means that do not share a letter are significantly different.*

**Tukey Pairwise Comparisons: Senario*Station*Hour**

**Grouping Information Using the Tukey Method and 95% Confidence**

| **Senario*Station*Hour** | **N** | **Mean** | **Grouping** | | | | | | | | | |
| --- | --- | --- | --- | --- | --- | --- | --- | --- | --- | --- | --- | --- |
| Int. 3 4 | 3 | 1.81333 | A |  |  |  |  |  |  |  |  |  |
| Int. 3 3 | 3 | 1.75000 | A | B |  |  |  |  |  |  |  |  |
| Ext. 3 4 | 3 | 1.64667 |  | B | C |  |  |  |  |  |  |  |
| Ext. 3 3 | 3 | 1.62333 |  |  | C | D |  |  |  |  |  |  |
| Ext. 3 2 | 3 | 1.57667 |  |  | C | D | E |  |  |  |  |  |
| non 3 4 | 3 | 1.56000 |  |  | C | D | E | F |  |  |  |  |
| Int. 3 2 | 3 | 1.54333 |  |  | C | D | E | F |  |  |  |  |
| non 3 3 | 3 | 1.52333 |  |  |  | D | E | F |  |  |  |  |
| Ext. 3 1 | 3 | 1.52000 |  |  |  | D | E | F |  |  |  |  |
| Int. 3 1 | 3 | 1.48000 |  |  |  |  | E | F |  |  |  |  |
| non 3 1 | 3 | 1.47000 |  |  |  |  | E | F |  |  |  |  |
| non 3 2 | 3 | 1.45333 |  |  |  |  |  | F |  |  |  |  |
| non 2 4 | 3 | 0.94667 |  |  |  |  |  |  | G |  |  |  |
| Ext. 2 4 | 3 | 0.94000 |  |  |  |  |  |  | G |  |  |  |
| non 2 3 | 3 | 0.92667 |  |  |  |  |  |  | G |  |  |  |
| non 2 1 | 3 | 0.88333 |  |  |  |  |  |  | G | H |  |  |
| Ext. 2 3 | 3 | 0.87667 |  |  |  |  |  |  | G | H |  |  |
| non 2 2 | 3 | 0.87667 |  |  |  |  |  |  | G | H |  |  |
| Int. 2 4 | 3 | 0.80667 |  |  |  |  |  |  |  | H | I |  |
| Ext. 2 2 | 3 | 0.79833 |  |  |  |  |  |  |  | H | I |  |
| Int. 2 3 | 3 | 0.78000 |  |  |  |  |  |  |  | H | I |  |
| Ext. 2 1 | 3 | 0.76333 |  |  |  |  |  |  |  |  | I |  |
| Int. 2 2 | 3 | 0.74333 |  |  |  |  |  |  |  |  | I |  |
| Int. 2 1 | 3 | 0.74000 |  |  |  |  |  |  |  |  | I |  |
| Int. 1 4 | 3 | 0.40333 |  |  |  |  |  |  |  |  |  | J |
| Int. 1 3 | 3 | 0.39000 |  |  |  |  |  |  |  |  |  | J |
| Int. 1 2 | 3 | 0.37333 |  |  |  |  |  |  |  |  |  | J |
| Int. 1 1 | 3 | 0.37000 |  |  |  |  |  |  |  |  |  | J |
| Ext. 1 4 | 3 | 0.34333 |  |  |  |  |  |  |  |  |  | J |
| Ext. 1 3 | 3 | 0.34000 |  |  |  |  |  |  |  |  |  | J |
| non 1 4 | 3 | 0.33000 |  |  |  |  |  |  |  |  |  | J |
| Ext. 1 2 | 3 | 0.33000 |  |  |  |  |  |  |  |  |  | J |
| non 1 3 | 3 | 0.32667 |  |  |  |  |  |  |  |  |  | J |
| Ext. 1 1 | 3 | 0.31000 |  |  |  |  |  |  |  |  |  | J |
| non 1 2 | 3 | 0.30000 |  |  |  |  |  |  |  |  |  | J |
| non 1 1 | 3 | 0.30000 |  |  |  |  |  |  |  |  |  | J |

*Means that do not share a letter are significantly different.*

WORKSHEET 1

**Comparisons for PMV**

**Tukey Pairwise Comparisons: Senario**

**Grouping Information Using the Tukey Method and 95% Confidence**

| **Senario** | **N** | **Mean** | **Grouping** | | |
| --- | --- | --- | --- | --- | --- |
| Ext. | 36 | 5.03528 | A |  |  |
| non | 36 | 4.20472 |  | B |  |
| Int. | 36 | 3.28083 |  |  | C |

*Means that do not share a letter are significantly different.*

**Tukey Pairwise Comparisons: Station**

**Grouping Information Using the Tukey Method and 95% Confidence**

| **Station** | **N** | **Mean** | **Grouping** | | |
| --- | --- | --- | --- | --- | --- |
| 3 | 36 | 5.26583 | A |  |  |
| 2 | 36 | 4.74611 |  | B |  |
| 1 | 36 | 2.50889 |  |  | C |

*Means that do not share a letter are significantly different.*

**Tukey Pairwise Comparisons: Hour**

**Grouping Information Using the Tukey Method and 95% Confidence**

| **Hour** | **N** | **Mean** | **Grouping** | | |
| --- | --- | --- | --- | --- | --- |
| 3 | 27 | 5.59926 | A |  |  |
| 4 | 27 | 5.29593 | A |  |  |
| 2 | 27 | 4.01222 |  | B |  |
| 1 | 27 | 1.78704 |  |  | C |

*Means that do not share a letter are significantly different.*

**Tukey Pairwise Comparisons: Senario*Station**

**Grouping Information Using the Tukey Method and 95% Confidence**

| **Senario*Station** | **N** | **Mean** | **Grouping** | | | | | |
| --- | --- | --- | --- | --- | --- | --- | --- | --- |
| Ext. 3 | 12 | 5.97500 | A |  |  |  |  |  |
| Ext. 2 | 12 | 5.33500 | A | B |  |  |  |  |
| non 3 | 12 | 4.98000 |  | B | C |  |  |  |
| Int. 3 | 12 | 4.84250 |  | B | C |  |  |  |
| non 2 | 12 | 4.46250 |  | B | C | D |  |  |
| Int. 2 | 12 | 4.44083 |  |  | C | D |  |  |
| Ext. 1 | 12 | 3.79583 |  |  |  | D | E |  |
| non 1 | 12 | 3.17167 |  |  |  |  | E |  |
| Int. 1 | 12 | 0.55917 |  |  |  |  |  | F |

*Means that do not share a letter are significantly different.*

**Tukey Pairwise Comparisons: Senario*Hour**

**Grouping Information Using the Tukey Method and 95% Confidence**

| **Senario*Hour** | **N** | **Mean** | **Grouping** | | | | |
| --- | --- | --- | --- | --- | --- | --- | --- |
| Ext. 3 | 9 | 6.33889 | A |  |  |  |  |
| non 4 | 9 | 6.28667 | A |  |  |  |  |
| non 3 | 9 | 5.84000 | A | B |  |  |  |
| Ext. 2 | 9 | 5.79556 | A | B |  |  |  |
| Int. 4 | 9 | 4.81444 |  | B | C |  |  |
| Ext. 4 | 9 | 4.78667 |  | B | C |  |  |
| Int. 3 | 9 | 4.61889 |  |  | C |  |  |
| non 2 | 9 | 3.46444 |  |  |  | D |  |
| Ext. 1 | 9 | 3.22000 |  |  |  | D |  |
| Int. 2 | 9 | 2.77667 |  |  |  | D |  |
| non 1 | 9 | 1.22778 |  |  |  |  | E |
| Int. 1 | 9 | 0.91333 |  |  |  |  | E |

*Means that do not share a letter are significantly different.*

**Tukey Pairwise Comparisons: Station*Hour**

**Grouping Information Using the Tukey Method and 95% Confidence**

| **Station*Hour** | **N** | **Mean** | **Grouping** | | | | | | | |
| --- | --- | --- | --- | --- | --- | --- | --- | --- | --- | --- |
| 3 3 | 9 | 7.24333 | A |  |  |  |  |  |  |  |
| 3 4 | 9 | 6.87111 | A | B |  |  |  |  |  |  |
| 2 3 | 9 | 6.29778 | A | B |  |  |  |  |  |  |
| 2 4 | 9 | 5.95556 |  | B | C |  |  |  |  |  |
| 3 2 | 9 | 5.03000 |  |  | C | D |  |  |  |  |
| 2 2 | 9 | 4.56111 |  |  |  | D |  |  |  |  |
| 1 3 | 9 | 3.25667 |  |  |  |  | E |  |  |  |
| 1 4 | 9 | 3.06111 |  |  |  |  | E | F |  |  |
| 1 2 | 9 | 2.44556 |  |  |  |  | E | F | G |  |
| 2 1 | 9 | 2.17000 |  |  |  |  |  | F | G | H |
| 3 1 | 9 | 1.91889 |  |  |  |  |  |  | G | H |
| 1 1 | 9 | 1.27222 |  |  |  |  |  |  |  | H |

*Means that do not share a letter are significantly different.*

**Tukey Pairwise Comparisons: Senario*Station*Hour**

**Grouping Information Using the Tukey Method and 95% Confidence**

| **Senario*Station*Hour** | **N** | **Mean** | **Grouping** | | | | | | | | | | | | |
| --- | --- | --- | --- | --- | --- | --- | --- | --- | --- | --- | --- | --- | --- | --- | --- |
| Ext. 3 3 | 3 | 7.65000 | A |  |  |  |  |  |  |  |  |  |  |  |  |
| non 3 4 | 3 | 7.61667 | A |  |  |  |  |  |  |  |  |  |  |  |  |
| Int. 3 4 | 3 | 7.28667 | A |  |  |  |  |  |  |  |  |  |  |  |  |
| non 3 3 | 3 | 7.15333 | A | B |  |  |  |  |  |  |  |  |  |  |  |
| Ext. 3 2 | 3 | 7.09000 | A | B |  |  |  |  |  |  |  |  |  |  |  |
| Int. 3 3 | 3 | 6.92667 | A | B | C |  |  |  |  |  |  |  |  |  |  |
| Ext. 2 3 | 3 | 6.61333 | A | B | C | D |  |  |  |  |  |  |  |  |  |
| non 2 4 | 3 | 6.52000 | A | B | C | D |  |  |  |  |  |  |  |  |  |
| Int. 2 4 | 3 | 6.34667 | A | B | C | D | E |  |  |  |  |  |  |  |  |
| Int. 2 3 | 3 | 6.16000 | A | B | C | D | E | F |  |  |  |  |  |  |  |
| non 2 3 | 3 | 6.12000 | A | B | C | D | E | F |  |  |  |  |  |  |  |
| Ext. 2 2 | 3 | 6.07000 | A | B | C | D | E | F |  |  |  |  |  |  |  |
| Ext. 3 4 | 3 | 5.71000 | A | B | C | D | E | F | G |  |  |  |  |  |  |
| Ext. 2 4 | 3 | 5.00000 |  | B | C | D | E | F | G | H |  |  |  |  |  |
| Ext. 1 3 | 3 | 4.75333 |  |  | C | D | E | F | G | H | I |  |  |  |  |
| non 1 4 | 3 | 4.72333 |  |  |  | D | E | F | G | H | I | J |  |  |  |
| non 1 3 | 3 | 4.24667 |  |  |  |  | E | F | G | H | I | J |  |  |  |
| Ext. 1 2 | 3 | 4.22667 |  |  |  |  | E | F | G | H | I | J |  |  |  |
| Int. 3 2 | 3 | 4.00333 |  |  |  |  |  | F | G | H | I | J |  |  |  |
| non 3 2 | 3 | 3.99667 |  |  |  |  |  | F | G | H | I | J |  |  |  |
| Int. 2 2 | 3 | 3.85667 |  |  |  |  |  |  | G | H | I | J |  |  |  |
| non 2 2 | 3 | 3.75667 |  |  |  |  |  |  | G | H | I | J |  |  |  |
| Ext. 2 1 | 3 | 3.65667 |  |  |  |  |  |  | G | H | I | J |  |  |  |
| Ext. 1 4 | 3 | 3.65000 |  |  |  |  |  |  | G | H | I | J |  |  |  |
| Ext. 3 1 | 3 | 3.45000 |  |  |  |  |  |  |  | H | I | J | K |  |  |
| non 1 2 | 3 | 2.64000 |  |  |  |  |  |  |  |  | I | J | K | L |  |
| Ext. 1 1 | 3 | 2.55333 |  |  |  |  |  |  |  |  |  | J | K | L |  |
| non 2 1 | 3 | 1.45333 |  |  |  |  |  |  |  |  |  |  | K | L | M |
| Int. 2 1 | 3 | 1.40000 |  |  |  |  |  |  |  |  |  |  | K | L | M |
| non 3 1 | 3 | 1.15333 |  |  |  |  |  |  |  |  |  |  |  | L | M |
| Int. 3 1 | 3 | 1.15333 |  |  |  |  |  |  |  |  |  |  |  | L | M |
| non 1 1 | 3 | 1.07667 |  |  |  |  |  |  |  |  |  |  |  | L | M |
| Int. 1 4 | 3 | 0.81000 |  |  |  |  |  |  |  |  |  |  |  | L | M |
| Int. 1 3 | 3 | 0.77000 |  |  |  |  |  |  |  |  |  |  |  | L | M |
| Int. 1 2 | 3 | 0.47000 |  |  |  |  |  |  |  |  |  |  |  | L | M |
| Int. 1 1 | 3 | 0.18667 |  |  |  |  |  |  |  |  |  |  |  |  | M |

*Means that do not share a letter are significantly different.*

WORKSHEET 1

**Comparisons for LW**

**Tukey Pairwise Comparisons: Senario**

**Grouping Information Using the Tukey Method and 95% Confidence**

| **Senario** | **N** | **Mean** | **Grouping** | |
| --- | --- | --- | --- | --- |
| Int. | 36 | 191.033 | A |  |
| non | 36 | 161.670 |  | B |
| Ext. | 36 | 161.661 |  | B |

*Means that do not share a letter are significantly different.*

**Tukey Pairwise Comparisons: Station**

**Grouping Information Using the Tukey Method and 95% Confidence**

| **Station** | **N** | **Mean** | **Grouping** | | |
| --- | --- | --- | --- | --- | --- |
| 1 | 36 | 292.094 | A |  |  |
| 2 | 36 | 130.256 |  | B |  |
| 3 | 36 | 92.014 |  |  | C |

*Means that do not share a letter are significantly different.*

**Tukey Pairwise Comparisons: Senario*Station**

**Grouping Information Using the Tukey Method and 95% Confidence**

| **Senario*Station** | **N** | **Mean** | **Grouping** | | | | |
| --- | --- | --- | --- | --- | --- | --- | --- |
| Int. 1 | 12 | 341.942 | A |  |  |  |  |
| non 1 | 12 | 267.189 |  | B |  |  |  |
| Ext. 1 | 12 | 267.149 |  | B |  |  |  |
| Int. 2 | 12 | 144.162 |  |  | C |  |  |
| Ext. 2 | 12 | 123.306 |  |  |  | D |  |
| non 2 | 12 | 123.300 |  |  |  | D |  |
| Ext. 3 | 12 | 94.527 |  |  |  |  | E |
| non 3 | 12 | 94.521 |  |  |  |  | E |
| Int. 3 | 12 | 86.994 |  |  |  |  | E |

*Means that do not share a letter are significantly different.*

**Tukey Pairwise Comparisons: Station*Hour**

**Grouping Information Using the Tukey Method and 95% Confidence**

| **Station*Hour** | **N** | **Mean** | **Grouping** | | |
| --- | --- | --- | --- | --- | --- |
| 1 3 | 9 | 292.133 | A |  |  |
| 1 2 | 9 | 292.097 | A |  |  |
| 1 1 | 9 | 292.076 | A |  |  |
| 1 4 | 9 | 292.069 | A |  |  |
| 2 2 | 9 | 136.432 |  | B |  |
| 2 4 | 9 | 128.209 |  | B |  |
| 2 3 | 9 | 128.204 |  | B |  |
| 2 1 | 9 | 128.178 |  | B |  |
| 3 4 | 9 | 96.658 |  |  | C |
| 3 3 | 9 | 90.477 |  |  | C |
| 3 2 | 9 | 90.463 |  |  | C |
| 3 1 | 9 | 90.458 |  |  | C |

*Means that do not share a letter are significantly different.*

**Tukey Pairwise Comparisons: Senario*Station*Hour**

**Grouping Information Using the Tukey Method and 95% Confidence**

| **Senario*Station*Hour** | **N** | **Mean** | **Grouping** | | | | |
| --- | --- | --- | --- | --- | --- | --- | --- |
| Int. 1 4 | 3 | 341.990 | A |  |  |  |  |
| Int. 1 3 | 3 | 341.967 | A |  |  |  |  |
| Int. 1 2 | 3 | 341.913 | A |  |  |  |  |
| Int. 1 1 | 3 | 341.900 | A |  |  |  |  |
| Ext. 1 3 | 3 | 267.230 |  | B |  |  |  |
| non 1 4 | 3 | 267.223 |  | B |  |  |  |
| Ext. 1 2 | 3 | 267.207 |  | B |  |  |  |
| non 1 3 | 3 | 267.203 |  | B |  |  |  |
| non 1 2 | 3 | 267.170 |  | B |  |  |  |
| Ext. 1 1 | 3 | 267.167 |  | B |  |  |  |
| non 1 1 | 3 | 267.160 |  | B |  |  |  |
| Ext. 1 4 | 3 | 266.993 |  | B |  |  |  |
| Int. 2 2 | 3 | 162.700 |  |  | C |  |  |
| Int. 2 4 | 3 | 138.000 |  |  |  | D |  |
| Int. 2 3 | 3 | 137.987 |  |  |  | D |  |
| Int. 2 1 | 3 | 137.960 |  |  |  | D |  |
| non 2 4 | 3 | 123.320 |  |  |  | D |  |
| Ext. 2 3 | 3 | 123.320 |  |  |  | D |  |
| non 2 3 | 3 | 123.307 |  |  |  | D |  |
| Ext. 2 2 | 3 | 123.307 |  |  |  | D |  |
| Ext. 2 4 | 3 | 123.307 |  |  |  | D |  |
| non 2 2 | 3 | 123.290 |  |  |  | D |  |
| Ext. 2 1 | 3 | 123.290 |  |  |  | D |  |
| non 2 1 | 3 | 123.283 |  |  |  | D |  |
| Int. 3 4 | 3 | 100.913 |  |  |  |  | E |
| Ext. 3 3 | 3 | 94.540 |  |  |  |  | E |
| non 3 4 | 3 | 94.533 |  |  |  |  | E |
| Ext. 3 4 | 3 | 94.527 |  |  |  |  | E |
| Ext. 3 2 | 3 | 94.527 |  |  |  |  | E |
| non 3 3 | 3 | 94.527 |  |  |  |  | E |
| Ext. 3 1 | 3 | 94.513 |  |  |  |  | E |
| non 3 2 | 3 | 94.513 |  |  |  |  | E |
| non 3 1 | 3 | 94.510 |  |  |  |  | E |
| Int. 3 3 | 3 | 82.363 |  |  |  |  | E |
| Int. 3 1 | 3 | 82.350 |  |  |  |  | E |
| Int. 3 2 | 3 | 82.350 |  |  |  |  | E |

*Means that do not share a letter are significantly different.*

WORKSHEET 1

**Comparisons for MRT**

**Tukey Pairwise Comparisons: Senario**

**Grouping Information Using the Tukey Method and 95% Confidence**

| **Senario** | **N** | **Mean** | **Grouping** | |
| --- | --- | --- | --- | --- |
| Ext. | 36 | 325.517 | A |  |
| non | 36 | 320.495 |  | B |
| Int. | 36 | 318.472 |  | B |

*Means that do not share a letter are significantly different.*

**Tukey Pairwise Comparisons: Station**

**Grouping Information Using the Tukey Method and 95% Confidence**

| **Station** | **N** | **Mean** | **Grouping** | | |
| --- | --- | --- | --- | --- | --- |
| 3 | 36 | 328.330 | A |  |  |
| 2 | 36 | 325.358 |  | B |  |
| 1 | 36 | 310.796 |  |  | C |

*Means that do not share a letter are significantly different.*

**Tukey Pairwise Comparisons: Hour**

**Grouping Information Using the Tukey Method and 95% Confidence**

| **Hour** | **N** | **Mean** | **Grouping** | | | |
| --- | --- | --- | --- | --- | --- | --- |
| 3 | 27 | 332.059 | A |  |  |  |
| 4 | 27 | 327.511 |  | B |  |  |
| 2 | 27 | 322.416 |  |  | C |  |
| 1 | 27 | 303.993 |  |  |  | D |

*Means that do not share a letter are significantly different.*

**Tukey Pairwise Comparisons: Senario*Hour**

**Grouping Information Using the Tukey Method and 95% Confidence**

| **Senario*Hour** | **N** | **Mean** | **Grouping** | | |
| --- | --- | --- | --- | --- | --- |
| Ext. 3 | 9 | 333.744 | A |  |  |
| non 4 | 9 | 333.580 | A |  |  |
| non 3 | 9 | 332.983 | A |  |  |
| Ext. 2 | 9 | 332.248 | A |  |  |
| Int. 3 | 9 | 329.448 | A |  |  |
| Int. 4 | 9 | 329.141 | A |  |  |
| Ext. 4 | 9 | 319.811 |  | B |  |
| non 2 | 9 | 317.663 |  | B |  |
| Int. 2 | 9 | 317.336 |  | B |  |
| Ext. 1 | 9 | 316.263 |  | B |  |
| Int. 1 | 9 | 297.962 |  |  | C |
| non 1 | 9 | 297.753 |  |  | C |

*Means that do not share a letter are significantly different.*

**Tukey Pairwise Comparisons: Station*Hour**

**Grouping Information Using the Tukey Method and 95% Confidence**

| **Station*Hour** | **N** | **Mean** | **Grouping** | | | | | | |
| --- | --- | --- | --- | --- | --- | --- | --- | --- | --- |
| 3 3 | 9 | 342.356 | A |  |  |  |  |  |  |
| 2 3 | 9 | 337.713 | A | B |  |  |  |  |  |
| 3 4 | 9 | 336.806 | A | B | C |  |  |  |  |
| 2 4 | 9 | 331.926 |  | B | C | D |  |  |  |
| 3 2 | 9 | 329.073 |  |  | C | D |  |  |  |
| 2 2 | 9 | 327.193 |  |  |  | D |  |  |  |
| 1 3 | 9 | 316.107 |  |  |  |  | E |  |  |
| 1 4 | 9 | 313.801 |  |  |  |  | E |  |  |
| 1 2 | 9 | 310.980 |  |  |  |  | E | F |  |
| 3 1 | 9 | 305.086 |  |  |  |  |  | F | G |
| 2 1 | 9 | 304.599 |  |  |  |  |  | F | G |
| 1 1 | 9 | 302.294 |  |  |  |  |  |  | G |

*Means that do not share a letter are significantly different.*

**Tukey Pairwise Comparisons: Senario*Station*Hour**

**Grouping Information Using the Tukey Method and 95% Confidence**

| **Senario*Station*Hour** | **N** | **Mean** | **Grouping** | | | | | | |
| --- | --- | --- | --- | --- | --- | --- | --- | --- | --- |
| non 3 4 | 3 | 342.643 | A |  |  |  |  |  |  |
| Ext. 3 3 | 3 | 342.637 | A |  |  |  |  |  |  |
| non 3 3 | 3 | 342.390 | A |  |  |  |  |  |  |
| Int. 3 3 | 3 | 342.040 | A |  |  |  |  |  |  |
| Int. 3 4 | 3 | 342.030 | A |  |  |  |  |  |  |
| Ext. 3 2 | 3 | 341.620 | A |  |  |  |  |  |  |
| non 2 3 | 3 | 338.447 | A | B |  |  |  |  |  |
| Ext. 2 3 | 3 | 338.323 | A | B |  |  |  |  |  |
| non 2 4 | 3 | 337.847 | A | B | C |  |  |  |  |
| Ext. 2 2 | 3 | 337.143 | A | B | C | D |  |  |  |
| Int. 2 3 | 3 | 336.370 | A | B | C | D |  |  |  |
| Int. 2 4 | 3 | 335.587 | A | B | C | D |  |  |  |
| Ext. 3 4 | 3 | 325.743 | A | B | C | D | E |  |  |
| Int. 3 2 | 3 | 322.827 |  | B | C | D | E | F |  |
| non 3 2 | 3 | 322.773 |  | B | C | D | E | F |  |
| Ext. 2 4 | 3 | 322.343 |  | B | C | D | E | F |  |
| Int. 2 2 | 3 | 322.280 |  | B | C | D | E | F |  |
| non 2 2 | 3 | 322.157 |  | B | C | D | E | F |  |
| Ext. 3 1 | 3 | 320.700 |  |  | C | D | E | F |  |
| Ext. 2 1 | 3 | 320.380 |  |  |  | D | E | F |  |
| Ext. 1 3 | 3 | 320.273 |  |  |  | D | E | F |  |
| non 1 4 | 3 | 320.250 |  |  |  | D | E | F |  |
| non 1 3 | 3 | 318.113 |  |  |  |  | E | F |  |
| Ext. 1 2 | 3 | 317.980 |  |  |  |  | E | F |  |
| Ext. 1 4 | 3 | 311.347 |  |  |  |  | E | F | G |
| Int. 1 3 | 3 | 309.933 |  |  |  |  | E | F | G |
| Int. 1 4 | 3 | 309.807 |  |  |  |  | E | F | G |
| non 1 2 | 3 | 308.060 |  |  |  |  |  | F | G |
| Ext. 1 1 | 3 | 307.710 |  |  |  |  |  | F | G |
| Int. 1 2 | 3 | 306.900 |  |  |  |  |  | F | G |
| Int. 1 1 | 3 | 299.607 |  |  |  |  |  |  | G |
| non 1 1 | 3 | 299.567 |  |  |  |  |  |  | G |
| Int. 3 1 | 3 | 297.440 |  |  |  |  |  |  | G |
| non 3 1 | 3 | 297.117 |  |  |  |  |  |  | G |
| Int. 2 1 | 3 | 296.840 |  |  |  |  |  |  | G |
| non 2 1 | 3 | 296.577 |  |  |  |  |  |  | G |

*Means that do not share a letter are significantly different.*
